# Research Review: The effects of mindfulness‐based interventions on cognition and mental health in children and adolescents – a meta‐analysis of randomized controlled trials

**DOI:** 10.1111/jcpp.12980

**Published:** 2018-10-22

**Authors:** Darren L. Dunning, Kirsty Griffiths, Willem Kuyken, Catherine Crane, Lucy Foulkes, Jenna Parker, Tim Dalgleish

**Affiliations:** ^1^ Medical Research Council Cognition and Brain Sciences Unit University of Cambridge Cambridge UK; ^2^ Department of Psychiatry University of Oxford Oxford UK; ^3^ Institute of Cognitive Neuroscience University College London London UK; ^4^ Cambridgeshire and Peterborough NHS Foundation Trust Cambridge UK

**Keywords:** Mindfulness, meta‐analysis, intervention, adolescence, attention

## Abstract

**Background:**

Mindfulness based interventions (MBIs) are an increasingly popular way of attempting to improve the behavioural, cognitive and mental health outcomes of children and adolescents, though there is a suggestion that enthusiasm has moved ahead of the evidence base. Most evaluations of MBIs are either uncontrolled or nonrandomized trials. This meta‐analysis aims to establish the efficacy of MBIs for children and adolescents in studies that have adopted a randomized, controlled trial (RCT) design.

**Methods:**

A systematic literature search of RCTs of MBIs was conducted up to October 2017. Thirty‐three independent studies including 3,666 children and adolescents were included in random effects meta‐analyses with outcome measures categorized into cognitive, behavioural and emotional factors. Separate random effects meta‐analyses were completed for the seventeen studies (*n* = 1,762) that used an RCT design with an active control condition.

**Results:**

Across all RCTs we found significant positive effects of MBIs, relative to controls, for the outcome categories of Mindfulness, Executive Functioning, Attention, Depression, Anxiety/Stress and Negative Behaviours, with small effect sizes (Cohen's *d*), ranging from .16 to .30. However, when considering only those RCTs with active control groups, significant benefits of an MBI were restricted to the outcomes of Mindfulness (*d *=* *.42), Depression (*d *=* *.47) and Anxiety/Stress (*d *=* *.18) only.

**Conclusions:**

This meta‐analysis reinforces the efficacy of using MBIs for improving the mental health and wellbeing of youth as assessed using the gold standard RCT methodology. Future RCT evaluations should incorporate scaled‐up definitive trial designs to further evaluate the robustness of MBIs in youth, with an embedded focus on mechanisms of action.

## Introduction

Mindfulness has been defined as intentionally directing attention to present moment experiences with an attitude of curiosity and acceptance (Bishop et al., 2004). Individual differences in levels of ‘dispositional’ mindfulness can be reliably assessed in both adults (Brown & Ryan, 2003; Buchheld, Grossman, & Walach, 2001; Feldman, Hayes, Kumar, Greeson, & Laurenceau, 2004) and children/adolescents (Greco, Dew, & Baer, 2006; Lawlor, Schonert‐Reichl, Gadermann, & Zumbo, 2014), with higher levels associated with better functioning for a range of psychological and physical health outcomes (e.g. Baer, Smith, Hopkins, Krietemeyer, & Toney, 2006; Barnes & Lynn, 2010; Brown, Ryan, & Creswell, 2007).

Mindfulness skills can be augmented through training, and a range of Mindfulness‐Based Interventions (MBIs) have been developed to enhance these capacities (Kabat‐Zinn, 1982; Segal, Williams, & Teasdale, 2002). It is hypothesized that the enhancement of proximal skills trained by MBIs, such as nonjudgmental attention control, may have downstream effects on more distal outcomes such as improved behaviour or reduced symptoms of psychopathology.

Although the origins of mindfulness are rooted in Buddhist philosophy and date back around two and a half thousand years, the earliest example of a formalized MBI is Mindfulness‐Based Stress Reduction (MBSR; Kabat‐Zinn, 1982; Kabat‐Zinn, 1990). MBSR was developed to help individuals learn to cope with and manage illness, pain and stress. Based on the principles of MBSR, other MBIs emerged in the years that followed, such as Mindfulness‐Based Cognitive Therapy (MBCT; Segal et al., 2002), that focuses on helping to prevent the recurrence of depression. In general, research with adults suggests that MBIs have positive effects on both mental and physical heath (e.g., Grossman, Niemann, Schmidt, & Walach, 2003; Khoury et al., 2013), though there are methodological concerns about some past studies, in terms of insufficient construct validity of outcome measures used, intervention methodology, and how the data are interpreted.

More recently, focus has turned to the benefits of using MBIs with children and adolescents. There are a number of rationales for introducing mindfulness to young people, including enhancing core cognitive skills to support academic and social functioning (for a review see Weare, 2003). Indeed, childhood and adolescence may be a particularly valuable time to practice mindfulness as self‐regulation and executive functioning develop markedly across this period (e.g. Blakemore & Choudhury, 2006). In addition, adolescence is a vulnerable period for the onset of mental health problems, with around 50% of all mental illness appearing before the age of 14 (Kessler et al., 2005). Since mindfulness training has demonstrated efficacy in preventing depressive relapse in adults (see Kuyken et al., 2016 for an individual participant data analysis of randomized controlled trials), it is appropriate to explore whether MBIs could also prevent depression or improve mental health and wellbeing in young people.

However, enthusiasm for MBIs with children and adolescents has arguably moved ahead of the evidence base (Greenberg & Harris, 2012). There are careful guidelines about the development and evaluation of complex interventions from theory through to implementation (MRC, 2000, 2008). Early‐stage evaluations along this trajectory include case studies and case series, and uncontrolled or nonrandomized trials. Although helpful in identifying the likely efficacy of an intervention and in ironing out procedural uncertainties (Cooksey, 2006), such early‐stage studies should be regarded as preparation for randomized, controlled trial (RCT) evaluations which represent the current gold standard assessment approach for emerging interventions.

In the case of MBIs for children and young people, the process to date has rarely moved beyond early stage evaluations: only about 30% of current studies have randomized participants to condition and around 50% have used a comparison condition (see Felver, Celis‐de Hoyos, Tezanos, & Singh, 2016 for a review). The choice of comparison condition used is also an issue, typically these are passive, nonactive comparisons such as no intervention, teaching/treatment as usual or wait list that do not account for nonspecific aspects of training that might nonetheless affect performance, such as spending time with a new teacher or increased motivation in simply doing something different. Currently, only around 10% of studies have used an ‘active’ comparison condition. An active control condition in MBI studies should refer to something that might be expected to benefit its participants and that matches the MBI in all nonspecific factors. Importantly, it should not include mindfulness as an ‘active ingredient’ so that differences between the groups can be attributed to an absence or a presence of mindfulness (see MacCoon et al., 2012 for a discussion).

### Overview and limitations of previous meta‐analyses

Although using mindfulness to improve the emotional, behavioural and cognitive outcomes of young people is a nascent field, there have already been four notable meta‐analyses. However, none of these has disaggregated early stage non‐RCT evaluations from RCT data, nor distinguished between the types of control groups used. Zoogman and colleagues reviewed 20 controlled and uncontrolled studies published between 2004 and 2011 with participants aged between 6 and 21 years. Results showed that MBIs significantly improved psychological symptoms, and attention/mindfulness, with small to small‐to‐moderate effect sizes (Zoogman, Goldberg, Hoyt, & Miller, 2015). Zenner, Herrnleben‐Kurz, and Walach (2014) synthesized 24 studies conducted in schools before 2012 with participants aged 6–19 years. A significant benefit of MBIs was reported for measures of cognition, stress and resilience with effect sizes ranging from small‐to‐moderate to large. There was no significant evidence that MBIs were useful in reducing emotional problems.

The two more recent meta‐analyses are larger, reflecting the increased interest in the utility of MBIs for improving the lives of young people. Klingbeil et al. (2017) included separate analyses of controlled, (*k* = 33), and uncontrolled (*k* = 43) studies with participants aged 4–18 years. Outcome measures fell into two broad categories. Those related to the skills of mindfulness, attention, meta‐cognition/cognitive flexibility that the MBI was designed to train, and those related to putative distal outcomes of academic achievement and emotional and behavioural regulation that are proposed to shift downstream as a function of applying the trained skills in day‐to‐day life. MBIs were shown to lead to significant improvements across outcomes in all categories, in both controlled and uncontrolled studies. Effect sizes for the uncontrolled studies ranged from small to small‐to‐moderate. For the controlled studies, all effect sizes were in the small‐to‐moderate range (Klingbeil et al., 2017). Finally, Maynard, Solis, Miller, and Brendel (2017) reported data from 35 controlled and uncontrolled studies exploring a range of MBIs delivered in schools to participants aged 4–20 years. This meta‐analysis showed that the MBIs significantly improved cognitive and socioemotional skills (effect sizes were small) but not academic or behavioural outcomes.

As might be expected, there is considerable overlap of included studies in the previous meta‐analyses. For example 90% of the studies included in the Zoogman et al. (2015) review are also present in the meta‐analysis by Klingbeil et al. (2017). Furthermore, and probably due to the lack of data available at the time, the Zoogman meta‐analysis combined mindfulness and attention measures into a single category whereas Klingbeil separately analysed these outcomes. Likewise, about 60% of the studies included in the Zenner meta‐analysis were also included by Maynard et al. (2017). Despite the overlap in included studies, the outcomes are categorized differently across the meta‐analyses. The Zennner et al. meta‐analysis categorized the outcome measures into emotional problems, stress, and resilience, whereas Maynard et al.'s meta‐analysis categorized the outcomes into socioemotional skills, behaviour outcomes and academic outcomes. Each meta‐analysis therefore assesses broadly the same sets of outcome measures but classifies them differently to create categories of particular interest to the authors. Consequently, identifying patterns across previous meta‐analyses is difficult. From the evidence it appears that MBIs may be useful for improving mindfulness (Klingbeil et al., 2017; Zoogman et al., 2015) and cognition (Klingbeil et al., 2017; Maynard et al., 2017; Zenner et al., 2014), though evidence for improvements in outcomes such as emotional and behavioural functioning is equivocal (see Table S1 in the online Supporting Information section for effect sizes for previous meta‐analyses, broken down by category).

A problem with the extant meta‐analyses, however, is the conflation of gold‐standard RCT data with earlier‐phase evaluations of MBIs in youth. There are at least two specific issues here. First, some of the studies in Zoogman et al.'s (2015) analysis did not include comparison groups; these are essential to control for test‐retest effects (Dikmen, Heaton, Grant, & Temkin, 1999) and maturational changes. Second, the meta‐analyses that separately evaluated studies with control groups included studies that did not randomize participants to condition (Maynard et al., 2017; Zenner et al., 2014). Nonrandomized studies are unguarded against expectancy or placebo effects. They are also at a greater risk of sampling bias (e.g., allocating participants to condition based on their preferences, likely compliance or need), that undermines external validity. In addition, all previous meta‐analyses of MBIs with youth have neglected to separately analyse RCT outcomes for studies that use active control groups from those that have a nonactive comparison arm (for example, no intervention or wait list). Active control groups are useful to control for aforementioned nonspecific effects. In addition, the use of these groups is essential to mitigate the Hawthorne effect (McCarney et al., 2007), used here to represent the phenomenon that when participants know they are in a control condition they are also aware that they are not expected to show pre‐ to post‐test improvements. This may be particularly true for participants in wait‐list control groups who are inevitably aware that they are not participating in an active treatment or might be demoralized by their randomization outcome and may be wary of presenting as improved in case the MBI is not offered at the end of the wait period (Furukawa et al., 2014).

### The current meta‐analysis

The current state‐of‐the‐art in the development of MBIs for young people is characterized by an increasing number of small RCTs, either with or without active control conditions. On the trajectory of complex intervention development, such smaller RCTs can be conceptualized as pilot, feasibility or platform trials for larger‐scale definitive RCTs which are fully powered, draw on large representative samples, compare the MBI against a plausible active control condition (often reflective of current best practice) with medium‐ to long‐term follow‐up, use a manualized intervention with well‐trained practitioners, and use published protocols with clearly identified primary outcome(s). At present, in the context of MBIs for youth, no such definitive evaluations are available, although some are in progress (e.g. the Kuyken et al., 2017, protocol). In the absence of any definitive trials of MBIs with children and adolescents, meta‐analytic synthesis of studies that characterize the current state‐of the‐art is essential. That was the aim behind the current meta‐analysis, which focusses exclusively on RCTs with either passive (no intervention, usual practice, or wait list) comparison conditions or comparison where a structured alternative to the MBI is included. The studies to date with these structured comparison conditions have comprised either control interventions designed principally to take account of nonspecific factors (henceforth ‘attention placebo controls’) or, in a relatively small number of studies (*k* = 9), control interventions with active ingredients designed to drive change in one or more specified outcomes (henceforth ‘active intervention controls’). In all of the RCTs employing active comparisons (*k* = 17), whether they be attention placebo or active intervention controls, reported here, the stated hypotheses predicted superiority of the MBI over the active control arm for the specified outcomes.

We took a number of other study selection decisions designed to retain the focus on MBIs delivered to a high standard. Consequently, the present meta‐analysis only comprises studies in which the MBI is focused primarily on mindfulness practice that originated from an established program (e.g., MBSR), rather than those in which elements of practice are substantially combined with other activities (e.g., mindful yoga, mindful colouring). All included studies involved MBIs delivered face‐to‐face over a series of sessions by trained mindfulness instructors with participants aged 18 years or younger. The included outcome measures were categorized into measures of mindfulness, cognition (executive functioning and attention), behaviour (social and negative behaviour) and emotion (depression and stress/anxiety) outcomes. Finally, moderator analyses to examine the importance of study quality (i.e., risk of bias), duration of MBI training (i.e., total number of hours of training) and the age of the participants included in the MBIs – likely to be a critical variable in youth studies – were also conducted to establish a) whether study quality effects results; b) whether the amount of time spent training drives the degree of improvement; and c) whether MBIs are particularly beneficial for younger children or older adolescents.

## Methods

The study was conducted in accordance with the Preferred Reporting Items for Systematic reviews and Meta‐Analyses (PRISMA) statement (Moher, Liberati, Tetzlaff & Altman, 2009) and was registered on the international prospective register of systematic reviews (PROSPERO), number 42016038364, on 13/05/2016.

### Search strategy and inclusion criteria

In October 2017, separate comprehensive literature searches for published and unpublished articles were carried out by two authors (DD, KG). Studies were identified from searches of keywords and titles in the electronic databases Pubmed, ERIC, Cochrane, EMBASE, PsycINFO, and Web of Science with the terms “mindful*” OR “MBCT” OR “MBSR” AND “child*” OR “school” OR “adolescen*” OR “youth” used (see Appendix S2 online in the Supporting Information section for the full set of search terms). We also checked reference lists of studies and reviews for additional potentially relevant studies. Prominent authors of mindfulness studies were contacted to ascertain if they had any unpublished data. No language or other limitations were imposed at this stage. The searches were then collated and, after duplicates were removed, the abstracts of the remaining studies were independently reviewed (DD, KG). If the abstract suggested that the study may be appropriate for inclusion in the meta‐analysis then the full‐text article of the study was evaluated against our inclusion criteria. These are as follows:

1 Study design: the effects of mindfulness were compared against a control condition (either no contact, waitlist, active or attention placebo control) and the participants were randomly assigned to condition;
2 Participants: the participants were aged 18 years or younger;
3 Intervention I: The core of the mindfulness training program consisted of the essential elements laid out by Crane et al., 2017 including:
  - Present moment focus and decentring;
  - The development of greater attentional and behavioural self‐regulation;
  - Engaging the participant in sustained mindfulness meditation practice
4 Intervention II:
  - The MBI was delivered over more than one session
  - Mindfulness practice was the central component of the intervention, rather than it being substantially combined with another activity (e.g., mindful yoga, mindful colouring) or a subcomponent of a broader complex intervention (e.g., Acceptance Commitment Therapy);
  - The mindfulness intervention was delivered by a trained mindfulness teacher;
5 Outcome variables: The outcome measures provided quantitative data from which effect sizes could be calculated. If the paper did not provide this then the authors were contacted.

**Figure 1:**
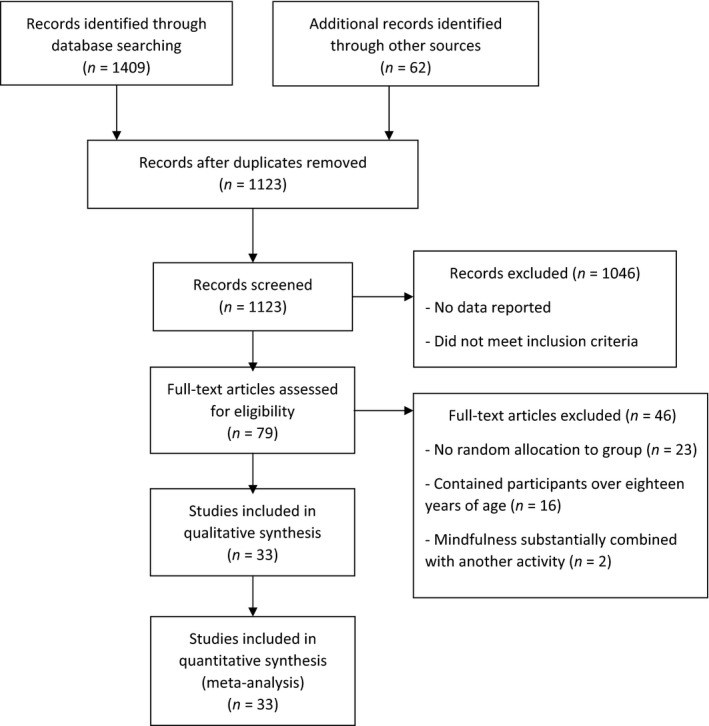
Flow diagram of literature search and study inclusion criteria

Thirty‐three studies met these inclusion criteria and were synthesized in the analysis (see Figure 1 for the PRISMA flow diagram). Table S3 (online supporting information) shows the studies included in the previous four meta‐analyses that were not included here and the reasons for exclusion.

We made the decision for our initial analysis to combine active intervention and attention placebo control groups under the single heading ‘active controls’. Likewise, the studies that used either no contact and wait‐list groups were combined and are known hereafter as ‘passive controls’. However, we do also present analyses by type of control group, see Table 3, and we return to this important issue in the Discussion.

### Data extraction and synthesis

For each study the following variables were recorded: age of sample, total number of participants in study (i.e., number in MBI group and number in control group), type of control condition(s), total hours of mindfulness training (i.e., sessions × duration in minutes, excluding home practice), total weeks of mindfulness training, type of mindfulness practice (e.g., MBSR) and the outcome measures reported. In cases where studies did not include all of these data, authors were contacted for more information.

Between‐group effect sizes (Cohen's *d*; Cohen, 1988) were calculated based on the mean prepost change in the MBI group minus the mean pre‐post change in the control group, divided by the pooled pretest standard deviation (Klauer, 2001; Morris, 2008). The pooled pretest standard deviation for weighting the differences in the pre‐post‐means was used so the intervention does not influence the standard deviation based on recommendation by Carlson and Schmidt (1999). A positive effect size indicates that the MBI group benefitted more than the comparison group. Effect sizes were interpreted on the following basis: *d *=* *.20, a small effect; *d *=* *.50, a moderate effect; and *d *=* *.80, a large effect (Cohen, 1988). For data synthesis, outcome measures were placed into one of the following categories: Mindfulness, Executive Functioning (e.g., planning, working memory, etc.), Attention, Depression, Stress/Anxiety, Negative behaviour (e.g., aggression, hostility, etc.) and Social behaviour (e.g., sharing, empathy, etc.). The decision to separate behavioural outcomes into negative and social behaviour was based on the view that these are dissociable constructs that could therefore respond differentially to an MBI. If studies used multiple measures to assess a single category then a mean of the effect sizes for these measures was calculated on pre‐ to postintervention effects. These categories were chosen based on those used in previous meta‐analyses and deemed most pertinent to children and adolescents. Outcome measures were placed into their chosen categories via discussion between all authors with disagreements decided through general consensus. A full list of the outcome measures used, their effect sizes and the categories in which they were placed can be seen in Table 4.

### Risk of bias

The Cochrane Collaboration's Risk of Bias Tool (Higgins & Green, 2011) was used to assess study quality. This involves making a series of evidence‐based judgements about specific features of each study to establish whether biases exist that could lead to an overestimation or underestimation of the true intervention effect. Two authors (DD, KG) independently graded the risk of bias in the following five categories: (a) random sequencing – if the method used to randomly allocate participants to group was appropriate; (b) allocation concealment – whether allocation to condition was concealed from participants during enrolment; (c) blinding of outcome assessment – if assessors were unaware of study condition; (d) incomplete outcome data – whether missing outcome data were appropriately reported and/or given appropriate statistical treatment; and (e) selective reporting – the degree to which studies reported all prespecified outcomes.

Each category for each study was given one of three ratings: a ‘minus’ indicated a low risk of bias; a ‘plus’ indicated a high risk of bias and a ‘question mark’ was used if the risk of bias was unclear (Higgins & Green, 2011). After all studies were independently rated, consensus was reached through discussion. To enable the use of risk of bias as a moderator of the effects of MBIs, a discrete variable was calculated. This was based on the following: each ‘plus’ was given a value of −1, each ‘question mark’ a value of 0 and each ‘minus’ a value of 1. Therefore, individual studies could have a risk of bias score of between −5 and 5, with lower scores indicating a higher risk of bias.

To investigate publication bias, the extent to which the studies included were representative of the population of studies, a series of Begg's funnel plots and Egger's regressions were conducted for each analysis. This is important for any meta‐analysis due to the tendency for journals to prefer to publish studies with positive rather than negative findings (Easterbrook, Gopalan, Berlin, & Matthews, 1991).

### Analysis

All analyses were conducted using version 3.3 of the Comprehensive Meta‐Analysis program (Borenstein, Hedges, Higgins, & Rothstein, 2005). Confidence intervals were calculated for effect sizes.

Heterogeneity, the amount of diversity in the characteristics of the outcome measures, was quantified using the *Q* statistic and *I* ^2^ estimates. For *I* ^2^ estimates, a value of 0% equates to no heterogeneity, 25% to low heterogeneity, 50% to moderate heterogeneity and 75% to high heterogeneity (Higgins & Green, 2011).

As a result of variation in studies (e.g., sample type, age of sample, outcome measures used) a random effects model was chosen for all analyses. All 33 studies were included in initial analyses and separate analyses were conducted on the 17 studies that used an active control condition. Individual, random‐effects meta‐regressions assessed the impact of our three identified moderator variables: age of sample, to establish if age determined who benefitted most from mindfulness; total hours of mindfulness training, to explore if dose of mindfulness training equates to better results; and risk of bias, to establish if the quality of study design impacted the results.

## Results

### All RCTs

Table 1 shows the results of the meta‐analysis for all 33 RCTs.

**Table 1:** Effect size analysis of RCT studies examining the efficacy of MBIs

	*k*	Number of effect sizes	Total *n*	Intervention effects			*z*	*p*	Heterogeneity			Publication bias (Eggers)	
				Mean effect size (*d*)	*SE*	95% CI			*Q* value	*p*	*I* ^2^ (%)	*t*	*p*
All measures	33	239	3,666	0.19	0.02	[.14 to .23]	8.65	<.01	790.07	<.01	69.75	4.37	<.01
Mindfulness	11	27	1,475	0.24	0.12	[.01 to .46]	2.06	.04	42.28	<.01	76.35	0.49	.64
Social behaviour	10	25	1,247	0.16	0.11	[−.05 to .37]	1.53	.13	30.95	<.01	67.69	0.83	.43
Negative behaviour	11	20	970	0.27	0.10	[.07 to .47]	2.60	<.01	21.00	.02	52.38	2.62	<.05
Depression	13	20	1,529	0.27	0.11	[.06 to .49]	2.53	<.01	44.36	<.01	72.95	2.85	<.05
Anxiety/Stress	20	41	2,319	0.16	0.06	[.04 to .27]	2.59	.01	40.21	<.01	47.78	2.19	<.05
Executive functions	15	25	1,691	0.30	0.09	[.12 to .49]	3.28	<.01	47.86	<.01	68.66	1.09	.30
Attention	8	8	1,158	0.19	0.08	[.04 to .34]	2.44	.02	10.78	.15	35.05	0.82	.42

Across all RCTs, those participants receiving an MBI improved significantly more than those receiving the control condition for the categories of Mindfulness and Executive Functions. The relative benefit of receiving MBIs for Attention was not significant. The categories of Depression and Anxiety/Stress showed significantly greater reductions after an MBI than after the control condition. MBIs did not have a significantly greater impact on changing Social Behaviour. However, the category of Negative Behaviour was significant, with MBI recipients showing a greater reduction in problems than those receiving the control condition. For statistically significant results, effect sizes ranged from small (.19) to small‐to‐moderate (.30).

### RCTs with active control groups

A subanalysis was conducted on only those 17 RCTs with active control groups (Table 2). These data show that those completing MBIs improved significantly more than those in active control interventions for Mindfulness and there was also a greater reduction in problems following an MBI than following the active control condition for the categories Depression and Anxiety/Stress. Effect sizes for significant results ranged from small (.18) to small‐to‐moderate (.42). There were no significant effects on changes in measures of Social Behaviour, Negative Behaviour, Executive Functions, or Attention.

**Table 2:** Effect size analysis of RCT studies with active control conditions examining the efficacy of MBIs

	*k*	Number of effect sizes	Total *n*	Intervention effects			*z*	*p*	Heterogeneity			Publication bias (Eggers)	
				Mean effect size (*d*)	*SE*	95% CI			*Q* value	*p*	*I* ^2^	*t*	*p*
All measures	17	141	1,762	0.20	0.03	[.14 to .26]	6.84	<.01	425.29	<.01	67.08	0.20	.83
Mindfulness	6	8	600	0.42	0.13	[.16 to .67]	3.23	<.01	9.07	.11	44.90	3.18	<.01
Social behaviour	6	18	708	−0.07	0.20	[−.46 to .31]	−0.38	.70	23.97	<.01	79.14	0.96	.39
Negative behaviour	5	15	580	0.22	0.19	[−.16 to .59]	1.13	.26	15.86	<.01	74.79	1.07	.36
Depression	6	11	520	0.47	0.13	[.22 to .72]	3.71	<.01	7.04	.22	28.92	2.06	.11
Anxiety/Stress	9	23	844	0.18	0.07	[.05 to .31]	2.65	<.01	4.21	.90	0.00	0.63	.55
Executive functions	7	12	958	0.10	0.07	[−.03 to .23]	1.49	.14	6.32	.39	5.10	0.01	.99
Attention	5	5	787	0.13	0.07	[−.01 to .28]	1.87	.06	2.93	.57	0.00	0.86	.45

### RCTs disaggregated by type of control group

Table 3 shows all RCTs disaggregated by control group type. MBIs showed significant benefits over all control group types with effect sizes ranging from small (.10) to small‐to‐moderate (.38).

**Table 3:** Meta‐analysis of effect sizes in favour of MBI across all outcome measures for RCTs disaggregated by type of control group used

	*k*	Number of effect sizes	Total *n*	Intervention effects			*z*	*p*	Heterogeneity		
				Mean effect size (*d*)	*SE*	95% CI			*Q* value	*p*	*I* ^2^
No contact	11	68	1,501	0.10	0.03	[.04 to .16]	3.03	<.01	209.32	<.01	67.99
Wait list	8	33	578	0.38	0.07	[.24 to .51]	5.45	<.01	83.11	<.01	61.50
Attention placebo	11	92	1,136	0.15	0.03	[.09 to .22]	4.65	<.01	263.47	<.01	65.46
Active intervention	9	45	813	0.26	0.06	[.15 to .37]	4.71	<.01	124.18	<.01	64.57

Gregoski, Barnes, Tingen, Harshfield, and Treiber (2011), Schonert‐Reichl et al. (2015), and Wright, Gregoski, Tingen, Barnes, and Treiber (2011) include both active and attention placebo controls; Atkinson and Wade (2015) include both no contact and active controls; Quach, Jastrowski Mano, and Alexander (2016) include both wait list and active controls.

### Heterogeneity

For all 33 RCTs, *Q* values show that there was a statistically significant level of heterogeneity for the categories of Mindfulness, Executive Functions, Negative Behaviour, Social Behaviour, and Depression. Significant scores ranged from 35.64 to 63.95. All evaluations of significant categories suggested a substantial amount of heterogeneity, with *I* ^2^ estimates ranging from 47.54% to 76.35% (Table 1).

For the 17 RCTs with active control groups, *Q* values showed that Negative Behaviour and Social Behaviour both showed a significant amount of heterogeneity with scores ranging from 15.86 to 29.18. *I* ^2^ estimates suggested that this was likely substantial in size, ranging from 74.79% to 79.14% (Table 2).

### Risk of bias

**Figure 2:**
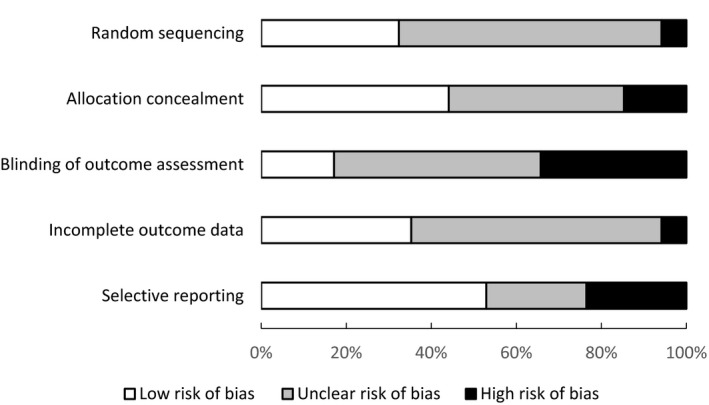
Risk of bias across all RCTs

Many authors failed to report key design characteristics to enable an accurate assessment of the risk of bias, even after being contacted for clarification. There was a low risk of bias in 32% of the studies for random sequencing, 44% for allocation concealment, 18% for blinding, 35% for incomplete outcome data and 53% for selective reporting. A high risk of bias existed in 6% of the studies for random sequencing, 15% for allocation concealment, 35% for blinding, 6% for incomplete outcome data and 24% for selective reporting. In all other cases the risk of bias was unclear (see Figure 2). For the risk of bias of individual studies, see Table 4.

**Table 4:** Details of the studies included in the meta‐analysis

Authors	Sample	*n* Mindfulness	*n* Controls	Control group	Age	Mindfulness training	Training period (weeks)	Total duration of training (hr)	Risk of bias				
Atkinson and Wade (2015)[Link]	General education (all female)	121	83	Passive – no contact	M = 15.7	Adapted from MBCT for depression	3	2.17	−	+	+	?	+
			96	Active – dissonance‐based training									
Barnes, Davis, Murzynowski, and Treiber (2004)[Link]	General education	34	39	Active – Health Education	M = 12.2	Breathing Awareness Meditation	12	14.00	?	−	?	?	−
Barnes, Pendergrast, Harshfield, and Treiber (2008)[Link]	General education (all classified as African American or Black)	17	39	Active – Health Education	M = 15.0	Breathing Awareness Meditation	12	14.00	?	−	?	?	−
Barnes, Gregoski, Tingen, and Treiber (2010)	General education	18	22	Active – Health Education	M = 16.2	Mindfulness‐based Eating Awareness	12	18.00	?	−	?	?	−
Biegel, Brown, Shapiro, and Schubert (2009)[Link]	A mix of mental health disorders	39	46	Passive – no contact	M = 15.7	MBSR	8	16.00	−	−	−	−	+
Bluth et al. (2015)[Link]	Low academic performance	14	13	Attention placebo – Substance abuse control	M = 16.8	Learning to Breathe	Not stated	6.00	−	−	?	?	−
Britton et al. (2014)[Link]	General education	52	48	Attention placebo – Asian history course	M = 11.8	Integrative Comtemplative Pedagogy	6	6.00	−	−	?	−	−
Desmond and Hanich (2010)[Link]	Minority, low income	15	25	Passive – no contact	M = 11.5	Mindful Awareness Practice	10	5.83	+	?	?	?	−
Flook et al. (2010)[Link]	General education	32	32	Attention placebo – Silent reading	M = 8.2	Mindful Awareness Practice	8	8.00	?	+	+	−	−
Flook, Goldberg, Pinger, and Davidson (2015)[Link]	General education	29	37	Passive – wait list	M = 4.7	Mindfulness Kindness curriculum	12	10.00	?	?	+	+	−
Franco, Mañas, Cangas, and Gallego (2010)[Link]	General education	31	30	Passive – wait list	M = 16.8	Meditacion Fluir	10	15.00	?	+	?	?	−
Franco Justo (2009)[Link]	General education	30	30	Passive – no contact	M = 17.3	Meditacion Fluir	10	15.00	?	?	−	?	−
Gregoski et al. (2011)[Link]	General education (all classified as African American or Black)	53	69	Attention placebo – Life Skills	M = 15.0	Breathing Awareness Meditation	12	14.00	?	−	?	−	−
			44	Active – Health Education									
Himelstein, Saul, and Garcia‐Romeu (2015)[Link]	Incarcerated males	14	13	Passive – no contact	M = 16.5	Mindfulness‐based substance abuse treatment	12	3.00	?	?	+	?	−
Johnson et al. (2016)	General education	115	154	Passive – no contact	M = 13.6	Dot be	9	6.00	−	+	+	−	−
Johnson et al. (2017)	General education	169	151	Passive – no contact	M = 13.4	Dot be	9	6.00	−	−	+	?	?
Johnson et al. (2017)	General education	179				Dot be w/parental involvement	9	6.00	−	−	+	?	?
Langer, Schmidt, Aguilar‐Parra, Cid, and Magni (2017)	General education	41	47	Passive – wait list	M = 13.4	MBCT/MBSR	8	6.00	?	?	?	−	?
Leonard et al. (2013)[Link]	Incarcerated males	147	117	Attention placebo – Cognitive‐perception intervention	M = 17.4	Power Source (elements of CBT)	4	12.50	?	?	?	?	−
Liehr and Diaz (2010)[Link]	Minority, low income	9	9	Attention placebo – Health Education	M = 9.5	Designed by Mindfulness in Schools	2	2.50	?	?	?	?	?
Napoli, Krech, and Holley (2005)[Link]	General education	97	97	Passive – no contact	R = 7–10	Attention Academy Program	24	9.00	?	?	?	?	+
Parker et al. (2014)[Link]	General education	71	40	Passive – wait list	M = 10.1	Master Mind	4	5.00	?	?	+	−	?
Poehlmann‐Tynan et al. (2016)[Link]	Economically disadvantaged	12	12	Attention placebo – Dialogic Reading	M = 4.7	The Kindness Curriculum	12	10.00	?	−	−	?	−
Potek (2012)[Link]	General education	16	14	Passive – wait list	M = 15	Learning to Breathe	6	4.50	−	?	+	−	?
Quach et al. (2016)[Link]	General education	54	53	Passive – wait list	R = 12–17	MBSR	4	6.00	−	?	?	−	+
			65	Active – Hatha yoga									
Ricarte, Ros, Latorre, and Beltran (2015)[Link]	General education	45	45	Passive – wait list	M = 8.9	Mindfulness Emotional Intelligence Training	6	1.50	?	?	?	?	+
Schonert‐Reichl et al. (2015)[Link]	General education	48	51	Attention placebo/Active – Social responsibility program	M = 10.2	MindUP	12	9.00	+	?	+	+	+
Semple, Lee, Rosa, and Miller (2010)[Link]	General education	13	12	Passive – wait list	M = 10.5	MBCT‐C	12	18.00	?	−	−	?	+
Shirk, DePrince, Crisostomo, and Labus (2014)	Depressed	20	23	Passive – no contact	M = 15.3	Mindfulness CBT	12	Not stated	?	−	?	−	+
Shomaker et al. (2017)	Girls at risk of type II diabetes	17	16	Active – Cognitive‐behavioural	M = 15.0	Learning to Breathe	6	6.00	−	−	+	?	?
Sibinga et al. (2013)[Link]	General education (all male)	22	19	Attention placebo – Health education	M = 12.5	MBSR	12	10.00	−	−	?	−	−
Sibinga, Webb, Ghazarian, and Ellen (2015)[Link]	General education	141	159	Attention placebo – Health education	M = 12.0	MBSR	12	10.00	?	−	−	−	?
Tan and Martin (2015)[Link]	A mix of mental health disorders	43	37	Passive – no contact	M = 15.4	Taming the Adolescent Mind	5	7.50	?	?	?	?	−
Wright et al. (2011)[Link]	General education (all classified as African American or Black)	35	42	Attention placebo – Life Skills	M = 15.0	Breathing Awareness Meditation	12	14.00	?	−	−	?	−
			44	Active – Health Education									

The study has been included in at least one previous meta‐analysis; M, Mean; R, Range; For risk of bias, − = low risk of bias, + = high risk of bias, ? = unclear risk of bias on the following indices: random sequencing, allocation concealment, blinding of outcome assessment, incomplete outcome data and selective reporting.

### Publication bias

For all 33 RCTs, Egger's tests showed that publication bias was evident for Negative Behaviour and Anxiety/Stress (Table 1). For the 17 RCTs with active control groups there was evidence of publication bias in the Mindfulness category only (Table 2).

### Moderator analysis

For all 33 RCTs, individual, random effects meta‐regressions showed that age was a significant moderator of improvements in Executive Functions (*Q* = 5.60, *p* = .018), with larger effect sizes in favour of the MBI associated with older age. For duration of MBI, total training hours was a significant moderator of a reduction in Negative Behaviour (*Q* = 7.30, *p* = .007), with larger effect sizes related to more hours of training. Interestingly, Risk of bias score had no significant effect on any outcome category.

For the 17 RCTs with active control groups, age significantly moderated improvements in Negative Behaviour (*Q* = 5.27, *p* = .021), with larger effect sizes associated with younger age. Risk of bias score significantly moderated measures of Mindfulness, with larger effect sizes related to greater risk of bias (*Q* = 4.36, *p* = .037). Total hours of MBI training had no significant effect on any outcome category.

## Discussion

This is the first meta‐analysis where only data from RCTs that examine the effects of MBIs on improving the mental health, behaviour and cognition of young people aged 18 years or younger, are included. Including only studies that compare MBIs against a control condition means that outcomes are guarded against test–retest effects and the effects of maturational changes in young people as these will be common across the MBI and comparator conditions. In addition, only using studies that adopt a randomized design ensures that there should be no systematic differences between groups.

Furthermore, and novel to this meta‐analyses, we include subanalyses of RCTs that compare MBIs against active comparison conditions (both attention placebo controls and active intervention controls). The combined risk of bias across the constituent studies in this meta‐analysis also appears to be smaller than in previous reviews that included a broader range of studies (e.g., Maynard et al., 2017), although the true risk of bias remains unclear as many of the included studies fail to adequately report potential issues such as the methods used for random sequencing and blinding.

### All RCTs

When including all 33 RCTs, results showed that MBIs lead to significantly greater improvements in measures of Mindfulness, Executive Functions and Attention, relative to the control conditions. This is encouraging given that MBIs are hypothesized to operate through improvements in both mindfulness and cognitive processes that can have a downstream effect on arguably more distal mental health and wellbeing outcomes. Indeed, benefits of MBIs were also evident in these latter outcomes, with postintervention benefits on measures of Depression and Anxiety/Stress and Negative Behaviours. For all significant results, effect sizes were small. Indeed, when using the current more rigorous selection criteria of only including RCTs, the overall effect size here is .19 (Cohen, 1992), which is smaller than the average effect sizes reported in previous meta‐analyses (Zoogman et al., 2015 (es = .23); Zenner et al., 2014 (es = .41); Klingbeil et al., 2017 (es = .17 to .51); Maynard et al., 2017 (es = .14 to .27)). This suggests that by including studies with less methodological rigour (e.g., lack of randomization), there may have been an overestimation of the effects of MBIs in previous meta‐analyses (Savović et al. 2017).

Age significantly moderated the effects of MBIs on Executive Functions, with greater relative benefits following an MBI associated with older age. It is possible that older adolescents benefit more than younger children due to what Roeser and Pinela (2014) describe as the ‘window of opportunity’. Indeed, the period between 14–18 years is seen as a key time for mindfulness to be effective due to heightened brain plasticity (Giedd, 2008). Furthermore, this age window is also characterized by increases in self‐reflection, social‐perspective taking and a greater interest in understanding the self and others (Blakemore & Mills, 2014; Harter, 1999; Selman, 2003). More work is needed to explore possible age‐related effects on gains in executive functions, particularly in younger children. The only previous meta‐analysis to consider if age moderated the effects of MBIs showed nonsignificant effects, though this analysis was based on an amalgam of all outcome variables rather than focusing on individual outcome categories (Zoogman et al., 2015).

The dose of MBI significantly moderated Negative Behaviour with more training associated with fewer negative behaviours. The previous, meta‐analyses provided no support for dose as a moderator of effects, though once again moderation analyses were considered across all outcomes, rather than examining individual outcome categories. The present results are in line with previous studies with adults that have reported that MBIs of longer duration produce greater general benefits (e.g., Mathew, Whitford, Kenny, & Denson, 2010; Parsons, Crane, Parsons, Fjorback, & Kuyken, 2017).

### RCTs with active control groups

When analysing the 17 MBI studies that used an RCT methodology against an active comparison group, the only significant effects for the MBI relative to the control condition that were retained from the wider analysis on all 33 trials, were Mindfulness, Depression and Anxiety/Stress. For the Mindfulness and Depression categories, effect sizes were approaching moderate in size, and for the Anxiety/Stress category, the effect size was small. These results are consistent with previous meta‐analyses with adults (e.g. Khoury, Sharma, Rush, & Fournier, 2015; Khoury et al., 2013), which show that MBIs are particularly useful for improving measures of mindfulness, stress, depression and anxiety, with greater improvements in mindfulness associated with larger improvements in measures of mental health. However, it is important to note that the beneficial effect of an MBI on the category of Mindfulness relative to an active control is moderated by risk of bias such that studies with a higher risk of bias produced bigger effect sizes. This suggests that the results of less tightly controlled studies may be artificially inflating the overall effect size.

The categories of Negative Behaviour, Executive Functions and Attention no longer improved significantly following an MBI when with trials were restricted to those including an active control intervention. This perhaps indicates that studies with passive control groups may erroneously inflate the effects of an MBI on these outcome domains. Alternatively, it might be indicative of the smaller effect sizes expected when MBIs are compared against a true active intervention that trains the same targeted outcomes as MBIs and we discuss this further below.

Finally, although Negative Behaviour was not significantly reduced overall in the group of studies including an active control condition, MBIs effects on Negative Behaviour were significantly moderated by age. This time younger children showed greater improvements than older children/adolescents following an MBI. This suggests that, when using a more robust trial design, although there is a lack of support that MBIs reduce negative behaviour across the age range studied, it is conceivable that they may be useful in reducing negative behaviour in younger children (see Parker, Kupersmidt, Mathis, Scull, & Sims, 2014; Schonert‐Reichl et al., 2015). To test this, researchers might want to consider comparing how the negative behaviour of older and younger children compare following MBIs in large‐scale, tightly controlled RCTs.

### RCTs by specific control group type

When RCTs of MBIs are further disaggregated according to the specific type of control group used (e.g., wait‐list, no contact, attention placebo control and active intervention control conditions), the pattern of findings was unexpected. Although, wait‐list controls unsurprisingly yielded the largest mean effect size, the smallest (i.e., the smallest difference between MBI and controls) emerged when comparing MBIs against no contact control groups. Theoretically, comparing an MBI against a passive control condition, such as a no contact group, presents a greater opportunity for gains in the MBI than when comparing it against an attention placebo or active intervention control condition as the influence of both nonspecific aspects of training and the Hawthorn effect are minimized (McCarney et al., 2007). It is plausible here that disaggregating our studies into these four smaller categories renders the categories more susceptible to the influence of individual studies. Indeed, in this case it seems that the two large studies by Johnson, Burke, Brinkman, and Wade (2016, 2017) that compared an MBI against a no contact control group and that produced small or negative effect sizes could be driving the small effect size in this category.

In general, several of the control group types are somewhat underrepresented in the analysis. In particular wait‐list controls (33 effect sizes) and active intervention controls (45 effect sizes). This underrepresentation drove our decision to combine the no contact and wait‐list controls and attention placebo and active intervention controls for our primary analyses. As the field of MBIs grow, future meta‐analyses will need to revisit the important issue of control group type in relation to the size of effects.

### Limitations

When all RCTs are included in the analysis, almost all of the categories suffer from heterogeneity. The presence of heterogeneity is indicative of a lack of similarity between the included studies, in this case perhaps with regards to the methodology used (e.g., different control groups). For the 17 RCTs with active control groups, heterogeneity is less of an issue but is still significantly present in both of the behavioural categories.

There was also evidence of publication bias in the sub‐categories of Negative Behaviour and Anxiety/Stress and in addition, for the RCTs with active control groups, in the Mindfulness category. It is important to highlight this as it suggests that the studies included here, in the aforementioned categories at least, are systematically different to unpublished studies. Specifically, there is an odds ratio of 2.3 for preferred publication of positive results (Dubben & Beck‐Bornholdt, 2005) suggesting that there may be an overestimation of the positive effects of MBIs. With respect to publication bias, a search of unpublished manuscripts was completed but perhaps partly due to the relatively exacting nature of our inclusion criteria, only one of the unpublished studies identified was eligible for inclusion in this review (see Potek, 2012).

Even though RCTs are the gold standard research design, there are still relatively few mindfulness studies with children/adolescents that adopt them, testifying to how early along the intervention development trajectory MBIs for youth are (Medical Research Council, 2000, 2008). As a consequence, some of our categories are populated by as few as five studies, which differ from one another in terms of participant characteristics and the nature of the active control group employed, making it difficult to be confident about the robustness of some of the findings arising from these analyses. Future studies should, and most likely will as MBIs develop as an intervention, consider inclusion of active intervention control conditions in their design to provide the most stringent test of the efficacy or noninferiority of MBIs. Indeed, the decision to combine active intervention and attention placebo controls was due to the relatively low number of RCTs across these two types of trial design. Many standard definitions of control groups would view attention placebo controls, although useful for controlling for certain nonspecific effects of an intervention, as therapeutically inactive (Higgins & Green, 2011).

Another potential issue is that many studies choose to test new MBI protocols rather than run replication studies on established MBIs (see Felver et al., 2016 for a discussion). Indeed, of the 22 different MBI protocols included in this review, only seven were used in more than one study. Indeed, over 40% of the MBI protocols included here were developed and implemented by the researchers themselves, enhancing the likelihood of bias when those interventions are evaluated by their developers (see Leykin & DeRubeis, 2009, for a discussion of allegiance effects).

Fewer than a third of studies included a measure of mindfulness as an outcome even though it is presumably viewed as a core mechanism of change for MBIs. It is important for future studies to attempt to measure mechanisms of change including mindfulness in order to enhance our understanding of how MBIs might be working with respect to different categories of outcome.

The outcome variables for the studies included here were typically scores on self‐report measures. Although commonly used in psychological intervention research, self‐report measures by definition rely on participants giving answers that are honest rather than socially acceptable, modulated by study demand effects or designed to portray the respondent in a favourable light (Podsakoff & Organ, 1986). Future studies would clearly be strengthened if self‐report measures are augmented with observer‐rated measures and/or direct physiological or behavioural measures of relevant outcome variables.

Given that the original rationale for developing formal MBI protocols was to improve mental health (MBSR; Kabat‐Zinn, 1982; Kabat‐Zinn, 1990), the superiority of MBIs over active comparison conditions for these outcomes is reassuring. However, more work is needed that directly compares MBIs with psychological and psychosocial interventions aimed at reducing mental health problems or increasing wellbeing – i.e., RCTs with true active intervention controls – to provide a stronger test of the utility of MBIs in this area. These trials will not necessarily involve superiority trial designs. Demonstrating the noninferiority of an MBI for these health outcomes would be important given the putative benefits of MBIs for broader aspects of cognition and well‐being.

## Conclusion and future directions

This meta‐analysis is the first to synthesize studies that have used an RCT design to compare MBIs in young people to a control condition. In addition, this is the first meta‐analysis to include separate analyses of RCTs with active control groups. Across all RCTs we found support for significant effects of an MBI, relative to the comparison condition, for outcome categories of Mindfulness, Executive Functioning, Attention, Depression, Anxiety/Stress and Negative Behaviours. Across the 17 RCTs with active control groups, support for significant benefits of an MBI was restricted to the outcomes of Mindfulness, Depression and Anxiety/Stress. Effect sizes for these significant effects ranged from small to small‐to‐moderate. There was some indication that age and dose of MBI moderated outcomes in some categories.

Addressing the mental health needs of children and adolescents has become an increasing focus in schools (Carsley, Heath, & Fajnerova, 2015; McMartin, Kingsbury, Dykxhoorn, & Colman, 2014), with MBIs becoming a popular, and relatively cost‐effective method of offering support (e.g., Felver et al., 2016; Tan, 2015). This meta‐analysis reinforces the promise of using MBIs for improving the mental health and wellbeing of youth when using the gold standard RCT methodology. Future RCT evaluations should incorporate scaled‐up definitive trial designs to further evaluate the robustness of MBIs in youth (Medical Research Council, 2000, 2008), for example, the ongoing MYRIAD trial (Kuyken et al., 2017), with a focus on mechanisms of action to further enhance evolving MBI protocols.

**Key points**

- Mindfulness based interventions (MBIs) are a popular way of attempting to improve the mental and physical health outcomes of children and adolescents.
- This is the first meta‐analysis of MBIs with youth composed exclusively of randomized controlled trials (RCTs) including RCTs with active control groups – the gold standard in intervention studies.
- When using the gold standard research design results showed that MBIs are useful in improving Depression and Anxiety outcomes, but not behavioural or cognitive outcomes,
- The meta‐analysis advocates the use of MBIs for improving mental health in young people.
- Future RCT evaluations should incorporate scaled‐up definitive trial designs to further evaluate the robustness of MBIs in youth, with an embedded focus on mechanisms of action.

## Supporting information


**Table S1.** Effect sizes from previous meta‐analyses for outcome categories.

**Table S2.** A list of all effect sizes by study.

**Table S3.** List of excluded studies and reason(s) for exclusion.

**Appendix S1.** References for excluded studies.

**Appendix S2.** Search terms used in literature search.

## References

[jcpp12980-bib-0001] *Atkinson, M.J. , & Wade, T.D. (2015). Mindfulness‐based prevention for eating disorders: A school‐based cluster randomized controlled study. International Journal of Eating Disorders, 48, 1024–1037. 26052831 10.1002/eat.22416

[jcpp12980-bib-0002] Baer, R.A. , Smith, G.T. , Hopkins, J. , Krietemeyer, J. , & Toney, L. (2006). Using self‐report assessment methods to explore facets of mindfulness. Assessment, 13, 27–45. 16443717 10.1177/1073191105283504

[jcpp12980-bib-0003] *Barnes, V.A. , Davis, H.C. , Murzynowski, J.B. , & Treiber, F.A. (2004). Impact of meditation on resting and ambulatory blood pressure and heart rate in youth. Psychosomatic Medicine, 66, 909–914. 15564357 10.1097/01.psy.0000145902.91749.35

[jcpp12980-bib-0004] *Barnes, V.A. , Gregoski, M.J. , Tingen, M.S. , & Treiber, F.A. (2010). Influences of family environment and meditation efficacy on hemodynamic function among African American adolescents. Journal of Complementary and Integrative Medicine, 7, 13–26. 10.2202/1553-3840.1326 PMC3276075 22328869

[jcpp12980-bib-0005] Barnes, S.M. , & Lynn, S.J. (2010). Mindfulness skills and depressive symptoms: A longitudinal study. Imagination, Cognition and Personality, 30, 77–91.

[jcpp12980-bib-0006] *Barnes, V.A. , Pendergrast, R.A. , Harshfield, G.A. , & Treiber, F.A. (2008). Impact of breathing awareness meditation on ambulatory blood pressure and sodium handling in prehypertensive African American adolescents. Ethnicity & Disease, 18, 1–5. PMC3216041 18447091

[jcpp12980-bib-0007] *Biegel, G.M. , Brown, K.W. , Shapiro, S.L. , & Schubert, C.M. (2009). Mindfulness‐based stress reduction for the treatment of adolescent outpatients: A randomized clinical trial. Journal of Consulting and Clinical Psychology, 77, 855–866. 19803566 10.1037/a0016241

[jcpp12980-bib-0008] Bishop, S.R. , Lau, M. , Shapiro, S. , Carlson, L. , Anderson, N.D. , Carmody, J. , … & Devins, G. (2004). Mindfulness: A proposed operational definition. Clinical Psychology: Science and Practice, 11, 230–241.

[jcpp12980-bib-0009] Blakemore, S.J. , & Choudhury, S. (2006). Development of the adolescent brain: Implications for executive function and social cognition. Journal of Child Psychology and Psychiatry, 47, 296–312. 16492261 10.1111/j.1469-7610.2006.01611.x

[jcpp12980-bib-0010] Blakemore, S.J. , & Mills, K.L. (2014). Is adolescence a sensitive period for sociocultural processing? Annual Review of Psychology, 65, 187–207. 10.1146/annurev-psych-010213-115202 24016274

[jcpp12980-bib-0011] *Bluth, K. , Campo, R.A. , Pruteanu‐Malinici, S. , Reams, A. , Mullarkey, M. , & Broderick, P.C. (2015). A school‐based mindfulness pilot study for ethnically diverse at‐risk adolescents. Mindfulness, 7, 90–104. 27034729 10.1007/s12671-014-0376-1 PMC4809539

[jcpp12980-bib-0012] Borenstein, M. , Hedges, L. , Higgins, J. , & Rothstein, H. (2005). Comprehensive meta‐analysis version 2 (p. 104). Englewood, NJ: Biostat.

[jcpp12980-bib-0013] *Britton, W.B. , Lepp, N.E. , Niles, H.F. , Rocha, T. , Fisher, E. , & Gold, J.S. (2014). A randomized controlled pilot trial of classroom‐based mindfulnesss meditation compared to an active control condition in sixth grade children. Journal of School Psychology, 52, 263–278. 24930819 10.1016/j.jsp.2014.03.002 PMC4060047

[jcpp12980-bib-0014] Brown, K.W. , & Ryan, R.M. (2003). The benefits of being present: Mindfulness and its role in psychological well‐being. Journal of Personality and Social Psychology, 84, 822. 12703651 10.1037/0022-3514.84.4.822

[jcpp12980-bib-0015] Brown, K.W. , Ryan, R.M. , & Creswell, J.D. (2007). Mindfulness: Theoretical foundations and evidence for its salutary effects. Psychological Inquiry, 18, 211–237.

[jcpp12980-bib-0016] Buchheld, N. , Grossman, P. , & Walach, H. (2001). Measuring mindfulness in insight meditation (Vipassana) and meditation based psychotherapy: The development of the Freiburg Mindfulness Inventory (FMI). Journal for Meditation and Meditation Research, 1, 11–34.

[jcpp12980-bib-0017] Carlson, K.D. , & Schmidt, F.L. (1999). Impact of experimental design on effect size: Findings from the research literature on training. Journal of Applied Psychology, 84, 851.

[jcpp12980-bib-0018] Carsley, D. , Heath, N.L. , & Fajnerova, S. (2015). Effectiveness of a classroom mindfulness coloring activity for test anxiety in children. Journal of Applied School Psychology, 31, 239–255.

[jcpp12980-bib-0019] Cohen, J. (1988). The effect size index: d. Statistical Power Analysis for the Behavioral Sciences, 2, 284–288.

[jcpp12980-bib-0020] Cohen, J. (1992). A power primer. Psychological Bulletin, 112, 155. 19565683 10.1037//0033-2909.112.1.155

[jcpp12980-bib-0021] Cooksey, D. (2006). A review of UK health research funding. London: The Stationery Office.

[jcpp12980-bib-0022] Crane, R.S. , Brewer, J. , Feldman, C. , Kabat‐Zinn, J. , Santorelli, S. , Williams, J.M.G. , & Kuyken, W. (2017). What defines mindfulness‐based programs? The warp and the weft. Psychological Medicine, 47, 990–999. 28031068 10.1017/S0033291716003317

[jcpp12980-bib-0023] *Desmond, C.T. , & Hanich, L. (2010). The effects of mindful awareness teaching practices on the executive functions of students in an urban, low income middle school. Available from http://www.wellnessworksinschools.com/WWResearchReport2010.pdf.

[jcpp12980-bib-0024] Dikmen, S.S. , Heaton, R.K. , Grant, I. , & Temkin, N.R. (1999). Test–retest reliability and practice effects of expanded Halstead‐Reitan Neuropsychological Test Battery. Journal of the International Neuropsychological Society, 5, 346–356. 10349297

[jcpp12980-bib-0025] Dubben, H.H. , & Beck‐Bornholdt, H.P. (2005). Systematic review of publication bias in studies on publication bias. BMJ, 331, 433–434. 15937056 10.1136/bmj.38478.497164.F7 PMC1188109

[jcpp12980-bib-0026] Easterbrook, P.J. , Gopalan, R. , Berlin, J.A. , & Matthews, D.R. (1991). Publication bias in clinical research. The Lancet, 337, 867–872. 10.1016/0140-6736(91)90201-y 1672966

[jcpp12980-bib-0027] Feldman, G.C. , Hayes, A.M. , Kumar, S.M. , Greeson, J.M. , & Laurenceau, J.P. (2004). Development, factor structure, and initial validation of the Cognitive and Affective Mindfulness Scale. Unpublished manuscript.

[jcpp12980-bib-0028] Felver, J.C. , Celis‐de Hoyos, C.E. , Tezanos, K. , & Singh, N.N. (2016). A systematic review of mindfulness‐based interventions for youth in school settings. Mindfulness, 7, 34–45.

[jcpp12980-bib-0029] *Flook, L. , Goldberg, S.B. , Pinger, L. , & Davidson, R.J. (2015). Promoting prosocial behavior and self‐regulatory skills in children through a mindfulness‐based kindness curriculum. Developmental Psychology, 51, 44–51. 25383689 10.1037/a0038256 PMC4485612

[jcpp12980-bib-0030] *Flook, L. , Smalley, S.L. , Kitil, M.J. , Kaiser‐Greenland, S. , Locke, J. , Ishijima, E. , & Kasari, C. (2010). Effects of mindful awareness practices on executive functions in elementary school children. Journal of Applied School Psychology, 26, 70–95.

[jcpp12980-bib-0031] *Franco Justo, C. (2009). Effectos de un programa demeditación sobre los niveles de creadividad verbal sobre un grupo de almunos/as de bachillerato (Effects of a meditation program on verbal creative levels in a group of students in late secondary education). Suma Psicológica, 16, 113–120.

[jcpp12980-bib-0032] *Franco, C. , Mañas, I. , Cangas, A.J. , & Gallego, J. (2010). The applications of mindfulness with students of secondary school: Results on the academic performance, self‐concept and anxiety In Lytras M.D., De Pablos P.O., Ziderman A., Roulstone A., Maurer H. & Imber J.B. (Eds.), Knowledge management, information systems, e‐learning, and sustainability research (pp. 83–97). Berlin, Germany: Springer‐Verlag.

[jcpp12980-bib-0033] Furukawa, T.A. , Noma, H. , Caldwell, D.M. , Honyashiki, M. , Shinohara, K. , Imai, H. , … & Churchill, R. (2014). Waiting list may be a nocebo condition in psychotherapy trials: A contribution from network meta‐analysis. Acta Psychiatrica Scandinavica, 130, 181–192. 24697518 10.1111/acps.12275

[jcpp12980-bib-0034] Giedd, J.N. (2008). The teen brain: Insights from neuroimaging. Journal of Adolescent Health, 42, 335–343. 18346658 10.1016/j.jadohealth.2008.01.007

[jcpp12980-bib-0035] Greco, L.A. , Dew, S.E. , & Baer, S. (2006). Child acceptance and mindfulness measure (CAMM). *Acceptance and Commitment Therapy. Measures Package*, 143.

[jcpp12980-bib-0036] Greenberg, M.T. , & Harris, A.R. (2012). Nurturing mindfulness in children and youth: Current state of research. Child Development Perspectives, 6, 161–166.

[jcpp12980-bib-0037] *Gregoski, M.J. , Barnes, V.A. , Tingen, M.S. , Harshfield, G.A. , & Treiber, F.A. (2011). Breathing awareness meditation and life skills training programs influence upon ambulatory blood pressure and sodium excretion among African American adolescents. Journal of Adolescent Health, 48, 59–64. 21185525 10.1016/j.jadohealth.2010.05.019 PMC3026442

[jcpp12980-bib-0038] Grossman, P. , Niemann, L. , Schmidt, S. , & Walach, H. (2003). Mindfulness‐based stress reduction and health benefits: A meta‐analysis. Focus on Alternative and Complementary Therapies, 8, 500. 10.1016/S0022-3999(03)00573-7 15256293

[jcpp12980-bib-0039] Harter, S. (1999). The construction of the self: A developmental perspective. New York: Guilford Press.

[jcpp12980-bib-0040] Higgins J.P., & Green S. (Eds.) (2011). Cochrane handbook for systematic reviews of interventions (Vol. 4). New York: John Wiley & Sons.

[jcpp12980-bib-0041] *Himelstein, S. , Saul, S. , & Garcia‐Romeu, A. (2015). Does mindfulness meditation increase effectiveness of substance abuse treatment with incarcerated youth? A pilot randomized controlled trial. Mindfulness, 6, 1472–1480.

[jcpp12980-bib-0042] *Johnson, C. , Burke, C. , Brinkman, S. , & Wade, T. (2016). Effectiveness of a school‐based mindfulness program for transdiagnostic prevention in young adolescents. Behaviour Research and Therapy, 81, 1–11. 27054828 10.1016/j.brat.2016.03.002

[jcpp12980-bib-0043] *Johnson, C. , Burke, C. , Brinkman, S. , & Wade, T. (2017). A randomized controlled evaluation of a secondary school mindfulness program for early adolescents: Do we have the recipe right yet? Behaviour Research and Therapy, 99, 37–46. 28910673 10.1016/j.brat.2017.09.001

[jcpp12980-bib-0044] Kabat‐Zinn, J. (1982). An outpatient program in behavioral medicine for chronic pain patients based on the practice of mindfulness meditation: Theoretical considerations and preliminary results. General Hospital Psychiatry, 4, 33–47. 7042457 10.1016/0163-8343(82)90026-3

[jcpp12980-bib-0045] Kabat‐Zinn, J. (1990). Full catastrophe living: The program of the stress reduction clinic at the University of Massachusetts Medical Center. New York, NY: Delta.

[jcpp12980-bib-0046] Kessler, R.C. , Berglund, P. , Demler, O. , Jin, R. , Merikangas, K.R. , & Walters, E.E. (2005). Lifetime prevalence and age‐of‐onset distributions of DSM‐IV disorders in the National Comorbidity Survey Replication. Archives of General Psychiatry, 62, 593–602. 15939837 10.1001/archpsyc.62.6.593

[jcpp12980-bib-0047] Khoury, B. , Lecomte, T. , Fortin, G. , Masse, M. , Therien, P. , Bouchard, V. , … & Hofmann, S.G. (2013). Mindfulness‐based therapy: A comprehensive meta‐analysis. Clinical Psychology Review, 33, 763–771. 23796855 10.1016/j.cpr.2013.05.005

[jcpp12980-bib-0048] Khoury, B. , Sharma, M. , Rush, S.E. , & Fournier, C. (2015). Mindfulness‐based stress reduction for healthy individuals: A meta‐analysis. Journal of Psychosomatic Research, 78, 519–528. 25818837 10.1016/j.jpsychores.2015.03.009

[jcpp12980-bib-0049] Klauer K.J. (Ed.) (2001). Handbuch kognitives training. Göttingen, Germany: Hogrefe.

[jcpp12980-bib-0050] Klingbeil, D.A. , Renshaw, T.L. , Willenbrink, J.B. , Copek, R.A. , Chan, K.T. , Haddock, A. , … & Clifton, J. (2017). Mindfulness‐based interventions with youth: A comprehensive meta‐analysis of group‐design studies. Journal of School Psychology, 63, 77–103. 28633940 10.1016/j.jsp.2017.03.006

[jcpp12980-bib-0051] Kuyken, W. , Nuthall, E. , Byford, S. , Crane, C. , Dalgleish, T. , Ford, T. , … & Williams, J.M.G. (2017). The effectiveness and cost‐effectiveness of a mindfulness training programme in schools compared with normal school provision (MYRIAD): Study protocol for a randomised controlled trial. Trials, 18, 194. 28446223 10.1186/s13063-017-1917-4 PMC5406917

[jcpp12980-bib-0052] Kuyken, W. , Warren, F.C. , Taylor, R.S. , Whalley, B. , Crane, C. , Bondolfi, G. , … & Segal, Z. (2016). Efficacy of mindfulness‐based cognitive therapy in prevention of depressive relapse: An individual patient data meta‐analysis from randomized trials. JAMA Psychiatry, 73, 565–574. 27119968 10.1001/jamapsychiatry.2016.0076 PMC6640038

[jcpp12980-bib-0053] *Langer, Á.I. , Schmidt, C. , Aguilar‐Parra, J.M. , Cid, C. , & Magni, A. (2017). Effects of a mindfulness intervention in Chilean high schoolers. Revista Medica de Chile, 145, 476–482. 28748995 10.4067/S0034-98872017000400008

[jcpp12980-bib-0054] Lawlor, M.S. , Schonert‐Reichl, K.A. , Gadermann, A.M. , & Zumbo, B.D. (2014). A validation study of the mindful attention awareness scale adapted for children. Mindfulness, 5, 730–741.

[jcpp12980-bib-0055] *Leonard, N.R. , Jha, A.P. , Casarjian, B. , Goolsarran, M. , Garcia, C. , Cleland, C.M. , … & Massey, Z. (2013). Mindfulness training improves attentional task performance in incarcerated youth: A group randomized controlled intervention trial. Frontiers in Psychology, 4, 792. 24265621 10.3389/fpsyg.2013.00792 PMC3820955

[jcpp12980-bib-0056] Leykin, Y. , & DeRubeis, R.J. (2009). Allegiance in psychotherapy outcome research: Separating association from bias. Clinical Psychology: Science and Practice, 16, 54–65.

[jcpp12980-bib-0057] *Liehr, P. , & Diaz, N. (2010). A pilot study examining the effect of mindfulness on depression and anxiety of minority children. Archives of Psychiatric Nursing, 24, 69–71. 20117691 10.1016/j.apnu.2009.10.001

[jcpp12980-bib-0058] MacCoon, D.G. , Imel, Z.E. , Rosenkranz, M.A. , Sheftel, J.G. , Weng, H.Y. , Sullivan, J.C. , … & Lutz, A. (2012). The validation of an active control intervention for Mindfulness Based Stress Reduction (MBSR). Behaviour Research and Therapy, 50, 3–12. 22137364 10.1016/j.brat.2011.10.011 PMC3257026

[jcpp12980-bib-0059] Mathew, K.L. , Whitford, H.S. , Kenny, M.A. , & Denson, L.A. (2010). The long‐term effects of mindfulness‐based cognitive therapy as a relapse prevention treatment for major depressive disorder. Behavioural and Cognitive Psychotherapy, 38, 561–576. 20374671 10.1017/S135246581000010X

[jcpp12980-bib-0060] Maynard, B.R. , Solis, M. , Miller, V. , & Brendel, K.E. (2017). Mindfulness‐based interventions for improving cognition, academic achievement, behavior and socio‐emotional functioning of primary and secondary students. Campbell Systematic Reviews, 13, 1–147.

[jcpp12980-bib-0061] McCarney, R. , Warner, J. , Iliffe, S. , Van Haselen, R. , Griffin, M. , & Fisher, P. (2007). The Hawthorne Effect: A randomised, controlled trial. BMC Medical Research Methodology, 7, 30. 17608932 10.1186/1471-2288-7-30 PMC1936999

[jcpp12980-bib-0062] McMartin, S.E. , Kingsbury, M. , Dykxhoorn, J. , & Colman, I. (2014). Time trends in symptoms of mental illness in children and adolescents in Canada. Canadian Medical Association Journal, 186, E672–E678. 25367419 10.1503/cmaj.140064 PMC4259795

[jcpp12980-bib-0063] Medical Research Council (2000). A framework for development and evaluation of RCTs for complex interventions to improve health. London: Medical Research Council.

[jcpp12980-bib-0064] Medical Research Council (2008). Developing and evaluating complex evaluations: New guidance. London: Medical Research Council.

[jcpp12980-bib-0065] Moher, D. , Liberati, A. , Tetzlaff, J. , & Altman, D.G. (2009). Preferred reporting items for systematic reviews and meta‐analyses: the PRISMA statement. Annals of Internal Medicine, 151, 264–269. 19622511 10.7326/0003-4819-151-4-200908180-00135

[jcpp12980-bib-0066] Morris, S.B. (2008). Estimating effect sizes from pretest‐posttest‐control group designs. Organizational Research Methods, 11, 364–386.

[jcpp12980-bib-0067] *Napoli, M. , Krech, P.R. , & Holley, L.C. (2005). Mindfulness training for elementary school students: The Attention Academy. Journal of Applied School Psychology, 21, 99–109.

[jcpp12980-bib-0068] *Parker, A.E. , Kupersmidt, J.B. , Mathis, E.T. , Scull, T.M. , & Sims, C. (2014). The impact of mindfulness education on elementary school students: Evaluation of the mastermind program. Advances in School Mental Health Promotion, 7, 184–204. 27057208 10.1080/1754730X.2014.916497 PMC4821437

[jcpp12980-bib-0069] Parsons, C.E. , Crane, C. , Parsons, L.J. , Fjorback, L.O. , & Kuyken, W. (2017). Home practice in mindfulness‐based cognitive therapy and mindfulness‐based stress reduction: A systematic review and meta‐analysis of participants' mindfulness practice and its association with outcomes. Behaviour Research and Therapy, 95, 29–41. 28527330 10.1016/j.brat.2017.05.004 PMC5501725

[jcpp12980-bib-0070] Podsakoff, P.M. , & Organ, D.W. (1986). Self‐reports in organizational research: Problems and prospects. Journal of Management, 12, 531–544.

[jcpp12980-bib-0071] *Poehlmann‐Tynan, J. , Vigna, A.B. , Weymouth, L.A. , Gerstein, E.D. , Burnson, C. , Zabransky, M. , … & Zahn‐Waxler, C. (2016). A pilot study of contemplative practices with economically disadvantaged preschoolers: Children's empathetic and self‐regulatory behaviors. Mindfulness, 7, 46–58.

[jcpp12980-bib-0072] *Potek, R. (2012). Mindfulness as a school‐based prevention program and its effect on adolescent stress, anxiety and emotional regulation. (Doctoral thesis), New York University,USA.

[jcpp12980-bib-0073] *Quach, D. , Jastrowski Mano, K.E. , & Alexander, K. (2016). A randomized controlled trial examining the effect of mindfulness meditation on working memory capacity in adolescents. Journal of Adolescent Health, 58, 489–496. 26576819 10.1016/j.jadohealth.2015.09.024

[jcpp12980-bib-0074] *Ricarte, J.J. , Ros, L. , Latorre, J.M. , & Beltran, M.T. (2015). Mindfulness‐based intervention in a rural primary school: Effects on attention, concentration, and mood. International Journal of Cognitive Therapy, 8, 258–270.

[jcpp12980-bib-0075] Roeser, R.W. , & Pinela, C. (2014). Mindfulness and compassion training in adolescence: A developmental contemplative science perspective. New Directions for Student Leadership, 2014, 9–30. 10.1002/yd.20094 25100492

[jcpp12980-bib-0076] Savović, J. , Turner, R.M. , Mawdsley, D. , Jones, H.E. , Beynon, R. , Higgins, J.P. , & Sterne, J.A. (2017). Association between risk‐of‐bias assessments and results of randomized trials in cochrane reviews: The ROBES meta‐epidemiologic study. American Journal of Epidemiology, 187, 1113–1122. 10.1093/aje/kwx344 PMC5928453 29126260

[jcpp12980-bib-0077] *Schonert‐Reichl, K.A. , Oberle, E. , Lawlor, M.S. , Abbott, D. , Thomson, K. , Oberlander, T.F. , & Diamond, A. (2015). Enhancing cognitive and social emotional development through a simple‐to‐administer mindfulness‐based program for elementary school children: A randomized controlled trial. Developmental Psychology, 51, 52–66. 25546595 10.1037/a0038454 PMC4323355

[jcpp12980-bib-0078] Segal, Z.V. , Williams, J.M.G. , & Teasdale, J.D. (2002). Mindfulness‐based cognitive therapy for depression: A new approach to relapse prevention. New York: Guilford.

[jcpp12980-bib-0079] Selman, R.L. (2003). Promotion of Social Awareness: Powerful Lessons for the Partnership of Developmental Theory and classroom practice. New York: Russell Sage Foundation.

[jcpp12980-bib-0080] *Semple, R.J. , Lee, J. , Rosa, D. , & Miller, L.F. (2010). A randomized trial of mindfulness‐based cognitive therapy for children: Promoting mindful attention to enhance social‐emotional resiliency in children. Journal of Child and Family Studies, 19, 218–229.

[jcpp12980-bib-0081] *Shirk, S.R. , DePrince, A.P. , Crisostomo, P.S. , & Labus, J. (2014). Cognitive behavioral therapy for depressed adolescents exposed to interpersonal trauma: An initial effectiveness trial. Psychotherapy, 51, 167. 24377410 10.1037/a0034845

[jcpp12980-bib-0082] *Shomaker, L.B. , Bruggink, S. , Pivarunas, B. , Skoranski, A. , Foss, J. , Chaffin, E. , … & Broderick, P. (2017). Pilot randomized controlled trial of a mindfulness‐based group intervention in adolescent girls at risk for type 2 diabetes with depressive symptoms. Complementary Therapies in Medicine, 32, 66–74. 28619307 10.1016/j.ctim.2017.04.003 PMC5705100

[jcpp12980-bib-0083] *Sibinga, E.M. , Perry‐Parrish, C. , Chung, S.E. , Johnson, S.B. , Smith, M. , & Ellen, J.M. (2013). School‐based mindfulness instruction for urban male youth: A small randomized controlled trial. Preventive Medicine, 57, 799–801. 24029559 10.1016/j.ypmed.2013.08.027

[jcpp12980-bib-0084] *Sibinga, E.M. , Webb, L. , Ghazarian, S.R. , & Ellen, J.M. (2015). School‐based mindfulness instruction: An RCT. Pediatrics, 137, 1–8. 10.1542/peds.2015-2532 26684478

[jcpp12980-bib-0085] Tan, L.B. (2015). A critical review of adolescent mindfulness‐based programmes. Clinical Child Psychology and Psychiatry, 21, 193–207. 25810416 10.1177/1359104515577486

[jcpp12980-bib-0086] *Tan, L. , & Martin, G. (2015). Taming the adolescent mind: A randomised controlled trial examining clinical efficacy of an adolescent mindfulness‐based group programme. Child and Adolescent Mental Health, 20, 49–55. 10.1111/camh.12057 32680328

[jcpp12980-bib-0087] Weare, K. (2003). Developing the emotionally literate school. London: Sage.

[jcpp12980-bib-0088] *Wright, L.B. , Gregoski, M.J. , Tingen, M.S. , Barnes, V.A. , & Treiber, F.A. (2011). Impact of stress reduction interventions on hostility and ambulatory systolic blood pressure in African American adolescents. Journal of Black Psychology, 37, 210–233. 22485058 10.1177/0095798410380203 PMC3319013

[jcpp12980-bib-0089] Zenner, C. , Herrnleben‐Kurz, S. , & Walach, H. (2014). Mindfulness‐based interventions in schools—a systematic review and meta‐analysis. Frontiers in Psychology, 5, 603. 25071620 10.3389/fpsyg.2014.00603 PMC4075476

[jcpp12980-bib-0090] Zoogman, S. , Goldberg, S.B. , Hoyt, W.T. , & Miller, L. (2015). Mindfulness interventions with youth: A meta‐analysis. Mindfulness, 6, 290–302.

