# Supplementary material for: Research Review: The effects of mindfulness‐based interventions on cognition and mental health in children and adolescents – a meta‐analysis of randomized controlled trials
Source: J Child Psychol Psychiatry. 2018 Oct 22;60(3):244–58. doi: 10.1111/jcpp.12980 (PMC6546608; doi:10.1111/jcpp.12980)
Supplement: Supplementary file 4 — Appendix S1. References for excluded studies. [file JCPP-60-244-s004.docx]

**Appendix S1.** References for excluded studies.

Ames, C. S., Richardson, J., Payne, S., Smith, P., & Leigh, E. (2014). Innovations in practice: Mindfulness therapy for depression in adolescents. *Child and Adolescent Mental Health, 19*, 74–78.

Anand, U., & Sharma, M. P. (2011). Impact of a mindfulness-based stress reduction program on stress and well-being in adolescents: A study at a school setting. *Journal of Indian Association of Child Adolescent Mental Health, 7*, 73–97.

Bakosh, L. S. (2013). *Investigating the effects of a daily audio-guided mindfulness intervention for elementary school students and teachers* (Doctoral dissertation, Institute of Transpersonal Psychology).

Bakosh, L. S., Snow, R. M., Tobias, J. M., Houlihan, J. L., & Barbosa-Leiker, C. (2016). Maximizing mindful learning: mindful awareness intervention improves elementary school students’ quarterly grades. *Mindfulness*, *7*(1), 59-67.

Barnert, E. S., Himelstein, S., Herbert, S., Garcia-Romeu, A., & Chamberlain, L. J. (2014). Exploring an intensive meditation intervention for incarcerated youth. *Child and Adolescent Mental Health, 19*, 69–73.

Barnes, V. A., Davis, H. C., Murzynowski, J. B. & Trieber, F. A. (2004). Impact of meditation on resting and ambulatory blood pressure and heart rate in youth*. Psychosomatic Medicine, 66(6)*, 909-914.

Beauchemin, J., Hutchins, T. L., & Patterson, F. (2008). Mindfulness meditation may lessen anxiety, promote social skills, and improve academic performance among adolescents with learning difficulties. *Complementary Health Practice Review, 13*, 34–45.

Bei, B., Bryne, M. L., Ivens, C., Waloszek, J.,Woods, M. J., Dudgeon, P., ... Allen, N. B. (2013). Pilot study of a mindfulness-based, multi-components, in-school group sleep intervention in adolescent girls. *Early Intervention in Psychiatry, 7,* 213–220.

Bennett, K., & Dorjee, D. (2015). The impact of a mindfulness-based stress reduction course (MBSR) on well-being and academic attainment of sixth-form students. *Mindfulness, 7*, 105–114.

Bergen-Cico, D., Razza, R., & Timmins, A. (2015). Fostering self-regulation through curriculum infusion of mindful yoga: A pilot study of efficacy and feasibility. *Journal of Child and Family Studies, 24*, 3448–3461.

Berking, M., & Wupperman, P. (2012). Emotion regulation and mental health: recent findings, current challenges, and future directions. *Current Opinion in Psychiatry, 25(2)*, 128-134.

Biegel,G., & Brown,K.W. (2010). Assessing the efficacy of an adapted in- class mindfulness-based training program for school-age children: a pilot study. White Paper. Available on line at: http://www.mindfulschools.org/pdf/Mindful %20Schools%20Pilot%20Study%20Whitepaper.pdf

Black, D. S., & Fernando, R. (2013).Mindfulness training and classroom behavior among lower-income and ethnic minority school children. *Journal of Child and Family Studies, 23,* 1242–1246.

Bogels, S., Hoogstad, B., van Dun, L., de Schutter, S., & Restifo, K. (2008). Mindfulness training for adolescents with externalizing disorders and their parents*. Behavioural and Cognitive Psychotherapy 36(2)*, 193–209.

Broderick, P. C., & Metz, S. (2009). Learning to BREATHE: A pilot trial of a mindfulness curriculum for adolescents. *Advances in School Mental Health, 2*, 35–46.

de Bruin, E. I., Blom, R., Smit, F. M. A., van Steensel, F. J. A., & Boegels, S.M. (2015). MYmind: Mindfulness training for youngsters with autism spectrum disorders and their parents. *Autism, 19*, 906–914.

Campbell, A. J. (2015). *The Impact of a School Mindfulness Program on Adolescent Stress, Wellbeing, and Emotion Regulation, with Attachment as a Moderator* (Doctoral dissertation, The George Washington University).

Carboni, J. A., Roach, A. T., & Fredrick, L. D. (2013). Impact of mindfulness training on the behavior of elementary students with Attention-Deficit/Hyperactive Disorder. *Research in Human Development, 10(3)*, 234–251.

Carsley, D., Heath, N. L., & Fajnerova, S. (2015). Effectiveness of a classroom mindfulness coloring activity for test anxiety in children*. Journal of Applied School Psychology, 31*, 239–255.

Catani, C., Kohiladevy,M., Ruf, M., Schauer, E., Elbert, T., & Neuer, F. (2009). Treating children traumatized bywar and tsunami: A comparison between exposure therapy and meditation-relaxation in north east Sri Lanka. *BMC Psychiatry, 9*, 22–33.

Coholic, D. A., & Eys, M. (2016). Benefits of an arts-based mindfulness group intervention for vulnerable children. *Child and Adolescent Social Work Journal, 33*, 1–13.

Coholic, D., Eys,M., & Lougheed, S. (2012). Investigating the effectiveness of an arts-based and mindfulness-based group program for the improvement of resilience in children in need. *Journal of Child and Family Studies, 21,* 833–844.

Corbett, M. L. (2011). *The effect of a mindfulness meditation intervention on attention, affect, anxiety, mindfulness, and salivary cortisol in school aged children*. Florida Atlantic University.

Cotton, S., Luberto, C. M., Sears, R. W., Strawn, J. R., Stahl, L., Wasson, R. S., ... & Delbello, M. P. (2016). Mindfulness‐based cognitive therapy for youth with anxiety disorders at risk for bipolar disorder: A pilot trial. *Early intervention in psychiatry*, *10*(5), 426-434.

Dehghani, F., Amiri, S., Molavi, H., & Neshat-Doost, H. T. (2014). Effectiveness of mindfulness based cognitive therapy on female elementary students with generalized anxiety disorder. *International Journal of Psychology and Behavioral Research, 3*, 159–165.

Edwards, M., Adams, E. M.,Waldo, M., Hadfield, O. D., & Biegel, G. M. (2014). Effects of a mindfulness group on Latino adolescent students: Examining levels of perceived stress, mindfulness, self-compassion, and psychological symptoms. *The Journal for Specialists in Group Work, 39*, 145–163.

Fishbein, D., Miller, S., Herman-Stahl, M., Williams, J., Lavery, B., Markovitz, L., ... Johnson, M. (2016). Behavioral and psychophysiological effects of a yoga intervention on high-risk adolescents: A randomized controlled trial. *Journal of Child and Family Studies, 25*, 518–529.

Franco Justo, C., de la Fuente Arias, M., & Salvador Granados, M. (2011). Impact of a training program in full consciousness (mindfulness) in the measure of growth and personal self-realization. *Psicothema, 23(1)*, 58–65.

Freedenberg, V. A., Thomas, S. A., & Friedmann, E. (2015). A pilot study of a mindfulness based stress reduction program in adolescents with implantable cardioverter defibrillators or pacemakers*. Pediatric Cardiology, 36*, 786–795.

Frenkel, M.O., Georg, A., Plessner, H., & Holt, D.V. (in press). Erste Ergebnisse zur Achtsam keitinder Schule: “8-sam,”ein Training für Jugend liche [Initial results of the Mindfulness in Schools “8-sam” Training for Teenagers].

Gould, L. F., Dariotis, J. K., Mendelson, T., & Greenberg, M. T. (2012). A school-based mindfulness intervention for urban youth: Exploring moderators of intervention effects. *Journal of Community Psychology, 40*, 968–982.

Haden, S. C., Daly, L. A., & Hagins, M. (2014). A randomised controlled trial comparing the impact of yoga and physical education on the emotional and behavioral functioning of middle school children. *Focus on Alternative and Complementary Therapies, 19(3)*, 148–155.

Hagins, M., Haden, S. C., & Daly, L. A. (2013). A randomized controlled trial on the effects of yoga on stress reactivity in 6th Grade Students. *Evidence-Based Complementary and Alternative Medicine,* 2013.

Haydicky, J., Wiener, J., Badali, P., Milligan, K., & Ducharme, J. M. (2012). Evaluation of a mindfulness-based intervention for adolescents with learning disabilities and co-occurring ADHD and anxiety. *Mindfulness, 3*, 151–164.

Haydicky, J., Shecter, C., Wiener, J., & Ducharme, J.M. (2013). Evaluation of MBCT for adolescents with ADHD and their parents: Impact on individual and family functioning. *Journal of Child and Family Studies*, 1–19.

Hennelly, S.(2011). The Intermediate and Sustained Effects of Mindfulness Training in Adolescence. Unpublished master’s thesis, Oxford Brookes University, Oxford.

Hesse, T., Holmes, L. G., Kennedy-Overfelt, V., Kerr, L. M., & Giles, L. L. (2015). Mindfulness-based intervention for adolescents with recurrent headaches: A pilot feasibility study. Evidence-based Complementary and Alternative Medicine (Advance online publication) 10.1155/2015/508958

Himelstein, S., Hastings, A., Shapiro, S., & Heery, M. (2012). Mindfulness training for self-regulation and stress with incarcerated youth: A pilot study. *Probation Journal, 59*, 151–165.

Huppert, F. A., & Johnson, D. M. (2010). A controlled trial of mindfulness of training in schools; the importance of practice for an impact on well-being. *The Journal of Positive Psychology, 5*, 264–274.

Jee, S. H., Couderc, J. -P., Swanson, D., Gallegos, A., Hilliard, C., Blumkin, A., ... Heinert, S. (2015). A pilot randomized trial teaching mindfulness-based stress reduction to traumatized youth in foster care. *Complementary Therapies in Clinical Practice, 21*, 201–209.

Joyce, A., Etty-Leal, J., Zazryn, T., & Hamilton, A. (2010). Exploring a mindfulness meditation program on the mental health of upper primary children: A pilot study. *Advances in School Mental Health Promotion, 3*, 17–25.

Khalsa, S. B. S., Hickey-Schultz, L., Cohen, D., Steiner, N., & Cope, S. (2012). Evaluation of the mental health benefits of yoga in a secondary school: A preliminary randomized controlled trial. *Journal of Behavioral Health Services & Research, 39(1),* 80–90.

Klatt, M., Harpster, K., Browne, E., White, S., & Case-Smith, J. (2013). Feasibility and preliminary outcomes for Move-Into-Learning: An arts-based mindfulness classroom intervention. *The Journal of Positive Psychology, 8(3)*, 233–241.

Koenig, K. P., Buckley-Reen, A., & Garg, S. (2012). Efficacy of the get ready to learn yoga program among children with autism spectrum disorders: a pretest-posttest control group design. *American Journal of Occupational Therapy, 66(5),* 538-546.

Kuyken, W., Weare, K., Ukoumunne, O. C., Vicary, R., Motton, N., Burnett, R., … Huppert, F. (2013). Effectiveness of the Mindfulness in Schools Programme: non-randomised controlled feasibility study. *The British Journal of Psychiatry, 203(2),* 126–131.

Lagor, A. F.,Williams, D. J., Lerner, J. B., & McClure, K. S. (2013). Lessons learned from a mindfulness-based intervention with chronically ill youth. *Clinical Practice in Pediatric Psychology, 1,* 146–158.

Lau, N., & Hue, M. (2011). Preliminary outcomes of a mindfulness-based for Hong-Kong adolescents in schools: Well-being, stress, and depressive symptoms. *International Journal of Children's Spirituality, 16,* 315–330.

Le, T. N., & Proulx, J. (2015). Feasibility of mindfulness-based intervention for incarcerated mixed-ethnic Native Hawaiian/Pacific Islander youth. *Asian American Journal of Psychology, 6*, 181–189.

Lee, J., Semple, R. J., Rosa, D., & Miller, L. (2008). Mindfulness-based cognitive therapy for children: Results of a pilot study. *Journal of Cognitive Psychotherapy: An International Quarterly, 22*, 15–28.

Mai, R. (2010). *Teaching mindfulness to low-ses, urban adolescents: a mixed methods study of process and outcomes*(Doctoral dissertation, Doctoral dissertation, Available from UMI Dissertation Express (AAT 3426960), New York, NY).

Malboeuf-Hurtubise, C., Achille, M., Muise, L., Beauregard-Lacroix, R., Vadnais, M., & Lacourse, E. (2016). A mindfulness-based meditation pilot study: Lessons learned on acceptability and feasibility in adolescents with cancer. *Journal of Child and Family Studies, 25*, 1168–1177.

Mehta, S., Mehta, V., Mehta, S., Shah, D., Motiwala, A., Vardhan, J., ... & Mehta, D. (2011). Multimodal behavior program for ADHD incorporating yoga and implemented by high school volunteers: A pilot study. *ISRN Pediatrics, 2011*, 1-5.

Mehta, S., Shah, D., Shah, K., Mehta, S., Mehta, N., Mehta, V., … Mehta, D. (2012). Peer-mediated multimodal intervention program for the treatment of children with ADHD in India: One year follow up. *ISRN Pediatrics, 2012*, 1-8. 419168.

Mendleson, T., Greenberg, M. T., Dariotis, J. K., Gould, L. F., Rhoades, B. L., & Leaf, P. J. (2010). Feasibility and preliminary outcomes of a school-based mindfulness intervention for urban youth. *Journal of Abnormal Child Psychology, 38*, 985–994.

Metz, S. M., Frank, J. L., Reibel, D., Cantrell, T., Sanders, R., & Broderick, P. C. (2013). The effectiveness of the Learning to BREATHE program on adolescent emotion regulation. *Research in Human Development, 10*, 252–272.

Milani, A., Nikmanesh, Z., & Farnam, A. (2013). Effectiveness ofmindfulness-based cognitive therapy (MBCT) in reducing aggression of individuals at the juvenile correction and rehabilitation center*. International Journal of High Risk Behavior & Addiction, 2*, 126–131.

Noggle, J. J., Steiner, N. J., Minami, T., & Khalsa, S. B. S. (2012). Benefits of yoga for psychosocial well-being in a U. S. high school curriculum: A preliminary randomized controlled trial. *Journal of Developmental and Behavioral Pediatrics, 33(3),* 193–201.

van der Oord, S., Bogels, S. M., & Peijnenburg, D. (2012). The effectiveness of mindfulness training for children with ADHD and mindful parenting for their parents. *Journal of Child and Family Studies, 21*, 139–147.

Peck, H. L., Kehle, T. J., Bray, M. A., & Theodore, L. A. (2005). Yoga as an intervention for children with attention problems. *School Psychology Review, 34(3)*, 415-424.

Ponitz, C. C., McClelland, M. M., Matthews, J. S., & Morrison, F. J. (2009). A structured observation of behavioral self-regulation and its contribution to kindergarten outcomes. *Developmental psychology, 45(3)*, 605.

Powell, L. A., Gilchrist, M., Stapley, J., Lesley Powell, M. G., & Jacqueline, S. (2008). A journey of self-discovery: an intervention involving massage, yoga and relaxation for children with emotional and behavioral difficulties attending primary schools. *European Journal of Special Needs Education, 23,* 403–412.

Quach, D. (2014). *Differential effects of sitting meditation and hatha yoga on working memory, stress, anxiety, and mindfulness among adolescents in a school setting*. Alliant International University.

Quinn, P. D., & Fromme, K. (2010). Self-Regulation as a Protective Factor against Risky Drinking and Sexual Behavior. *Psychology of Addictive Behaviors : Journal of the Society of Psychologists in Addictive Behaviors, 24(3),* 376–385.

Raes, P., Griffith, J. W., Van der Gucht, K., & Williams, J. M. G. (2014). School-based prevention and reduction of depression in adolescents: A cluster-randomized controlled trial of a mindfulness group program. *Mindfulness, 5,* 477–486.

Ramadoss, R., & Bose, B. K. (2010). Transformative life skills: Pilot studies of a yoga model for reducing perceived stress and improving self-control in vulnerable youth. *International Journal of Yoga Therapy, 20*, 75-80.

Raveepatarakul, J., Suttiwan, P., Iamsupasit, S., & Mikulas,W. L. (2014). A mindfulness enhancement program for Thai 8- to 11-year old children: Effects on mindfulness and depression. *Journal of Health Research, 28*, 335–341.

Razza, R. A., Bergen-Cico, D., & Raymond, K. (2015). Enhancing preschoolers' self-regulation via mindful yoga. *Journal of Child and Family Studies, 24*, 372–385.

Ricard, R. L. , Lerma, E., & Heard, C. C. C. (2013). Piloting a Dialectical Behavioral Therapy (DBT) infused skills group in a Disciplinary Alternative Education Program. *The Journal for Specialists in Group Work, 38(4)*, 285-306

Richardson, M., Abraham, C., & Bond, R. (2012). Psychological correlates of university students' academic performance: a systematic review and meta-analysis. *Psychological bulletin, 138(2)*, 353.

Salustri, M. E. (2009). *Mindfulness-based stress reduction to improve well-being among adolescents in an alternative high school*. Hofstra University.

Schonert-Reichl, K. A., & Lawlor, M. S. (2010). The effects of a mindfulness-based education program on pre-and early adolescents’ well-being and social and emotional competence. *Mindfulness, 1(3),* 137–151.

Sektnan, M., McClelland, M. M., Acock, A., & Morrison, F. J. (2010). Relations between early family risk, children’s behavioral regulation, and academic achievement. *Early Childhood Research Quarterly, 25(4)*, 464–479.

Semple, R. J., Reid, E. F. G., & Miller, L. (2005). Treating anxiety with mindfulness: An open trial of mindfulness training for anxious children. *Journal of Cognitive Psychotherapy, 19(4)*, 379–392.

Sibinga, E. M. S., Kerrigan, D., Stewart, M., Johnson, K., Magyari, T., & Ellen, J. M. (2011). Mindfulness-based stress reduction for urban youth. *The Journal of Alternative and Complementary Medicine, 17*, 213–218.

Singh, N. N., Lancioni, G. E., Singh Joy, S. D., Winton, A. S. W., Sabaawi, M., Wahler, R. G., & Singh, J. (2007). Adolescents with conduct disorder can be mindful of their aggressive behavior. *Journal of Emotional and Behavioral Disorders, 15(1)*, 56–63.

Smith, B. H., Connington, A., McQuillin, S., & Crowder Bierman, L. (2014). Applying the deployment focused treatment development model to school-based yoga for elementary school students: Steps one and two. *Advances in School Mental Health Promotion, 7(3),* 140–155.

Steiner, N. J., Sidhu, T. K., Pop, P. G., Frenette, E. C., & Perrin, E. C. (2013). Yoga in an urban school for children with emotional and behavioral disorders: A feasibility study. *Journal of Child and Family Studies, 22(6)*, 815–826.

Tan, L., & Martin, G. (2013). Taming the adolescent mind: Preliminary report of a mindfulness-based psychological intervention for adolescents with clinical heterogeneous mental health diagnoses*. Clinical Child Psychology and Psychiatry, 18*, 300–312.

Tharaldsen, K. (2012). Mindful coping for adolescents: beneficial or confusing. *Advances in School Mental Health Promotion, 5(2)*, 105–124.

van de Weijer-Bergsma, E., Formsma, A. R., de Bruin, E. I., & Bögels, S. M. (2012). The effectiveness of mindfulness training on behavioral problems and attentional functioning in adolescents with ADHD. *Journal of Child and Family Studies, 21*, 775–787.

van deWeijer-Bergsma, E., Langenberg, G., Brandsma, R., Oort, F. J., & Bögels, S. M. (2014). The effectiveness of a school-based mindfulness training as a program to prevent stress in elementary school children. *Mindfulness, 5*, 238–248.

Viafora, D., Mathiesen, S., & Unsworth, S. (2015). Teaching mindfulness to middle school students and homeless youth in school classrooms. *Journal of Child and Family Studies, 24(5)*, 1179– 1191.

Vickery, C., & Dorjee, D. (2015). Mindfulness training in primary schools decreases negative affect and increases meta-cognition in children. *Frontiers in Psychology, 6*, 2025.

White, L. S. (2012). Reducing stress in school-age girls through mindful yoga. *Journal of Pediatric Health Care, 26(1)*, 45–56.

Wick, K. M. (2013). The effect of mindfulness meditation and lovingkindness meditation on academic performance among female at-risk high school students (Dissertation). Walden University, Ann Arbor. Retrieved from ProQuest Dissertations & Theses Full Text. (1284937721).

Wisner, B. L. (2008). *The impact of meditation as a cognitive-behavioral practice for alternative high school students*. The University of Texas at Austin.

Worth, D. E. (2013). *Mindfulness meditation and attention-deficit/hyperactivity disorder symptom reduction in middle school students*. Walden University.

Zahn, W. L. (2008). *The effects of Tai Chi Chuan on mindfulness, mood, and quality of life in adolescent girls*. ProQuest.

Zylowska, L., Ackerman, D. L., Yang, M. H., Futrell, J. L., Horton, N. L., Hale, S. T., ... Smalley, S. L. (2008). Mindfulness meditation training with adults and adolescents with ADHD: A feasibility study. *Journal of Attention Disorders, 11*, 737–746.
