# Supplementary material for: Research Review: The effects of mindfulness‐based interventions on cognition and mental health in children and adolescents – a meta‐analysis of randomized controlled trials
Source: J Child Psychol Psychiatry. 2018 Oct 22;60(3):244–58. doi: 10.1111/jcpp.12980 (PMC6546608; doi:10.1111/jcpp.12980)
Supplement: Supplementary file 5 — Appendix S2. Search terms used in literature search. [file JCPP-60-244-s005.docx]

**Appendix S2.** Search terms used in literature search.

("mindfulness"[MeSH Terms] OR "mindfulness"[All Fields]) AND ((youth[All Fields] OR youth'[All Fields] OR youth''[All Fields] OR youth'07[All Fields] OR youth'12[All Fields] OR youth's[All Fields] OR youth,[All Fields] OR youth1111youth[All Fields] OR youth2000[All Fields] OR youth2000'[All Fields] OR youth888cn[All Fields] OR youthaccess[All Fields] OR youthaids[All Fields] OR youthalive[All Fields] OR youthanavanh[All Fields] OR youthbet[All Fields] OR youthbeyondblue[All Fields] OR youthcare[All Fields] OR youthcare's[All Fields] OR youthcoalition[All Fields] OR youthcount[All Fields] OR youthdale[All Fields] OR youtheewang[All Fields] OR youthempoweredsolutions[All Fields] OR youthen[All Fields] OR youthened[All Fields] OR youthening[All Fields] OR youther[All Fields] OR youthevaluator[All Fields] OR youthfacts[All Fields] OR youthfocus[All Fields] OR youthfriends[All Fields] OR youthful[All Fields] OR youthful'[All Fields] OR youthfully[All Fields] OR youthfulness[All Fields] OR youthfulness'[All Fields] OR youthhealth[All Fields] OR youthhealthtalk[All Fields] OR youthhood[All Fields] OR youthist[All Fields] OR youthkitty[All Fields] OR youthless[All Fields] OR youthline[All Fields] OR youthliners[All Fields] OR youthlink[All Fields] OR youthmain[All Fields] OR youthmapping[All Fields] OR youthmd98[All Fields] OR youthmentalhealth[All Fields] OR youthmood[All Fields] OR youthnet[All Fields] OR youtho2[All Fields] OR youthonline[All Fields] OR youthpc80[All Fields] OR youthpc802[All Fields] OR youthph80[All Fields] OR youthphase[All Fields] OR youthphotovoice[All Fields] OR youthprise[All Fields] OR youthprise's[All Fields] OR youthpsychiatry[All Fields] OR youthrise[All Fields] OR youths[All Fields] OR youths'[All Fields] OR youths'family[All Fields] OR youths'perceived[All Fields] OR youths'symptom[All Fields] OR youthsafe[All Fields] OR youthsave[All Fields] OR youthscan[All Fields] OR youthscene[All Fields] OR youthspace[All Fields] OR youthsportsortho[All Fields] OR youthsporttrust[All Fields] OR youthstyles[All Fields] OR youthwomen's[All Fields] OR youthwork[All Fields] OR youthzhw[All Fields]) OR (child[All Fields] OR child'[All Fields] OR child''[All Fields] OR child'head[All Fields] OR child'ren[All Fields] OR child's[All Fields] OR child's'[All Fields] OR child'shealth[All Fields] OR child'stalk[All Fields] OR child,[All Fields] OR child2[All Fields] OR child2015[All Fields] OR child317[All Fields] OR child3209[All Fields] OR childa[All Fields] OR childacute[All Fields] OR childadolescent[All Fields] OR childaeva[All Fields] OR childage[All Fields] OR childago[All Fields] OR childand[All Fields] OR childas[All Fields] OR childattention[All Fields] OR childbaring[All Fields] OR childbase[All Fields] OR childbearer[All Fields] OR childbearers[All Fields] OR childbearers'[All Fields] OR childbearimg[All Fields] OR childbearing[All Fields] OR childbearing'[All Fields] OR childbearing's[All Fields] OR childbearingage[All Fields] OR childbed[All Fields] OR childbeds[All Fields] OR childbirt[All Fields] OR childbirth[All Fields] OR childbirth'[All Fields] OR childbirth''[All Fields] OR childbirthconnection[All Fields] OR childbirthing[All Fields] OR childbirthis[All Fields] OR childbirths[All Fields] OR childbirthwas[All Fields] OR childblains[All Fields] OR childbood[All Fields] OR childbrain[All Fields] OR childbred[All Fields] OR childbreeding[All Fields] OR childbrith[All Fields] OR childcan[All Fields] OR childcar[All Fields] OR childcardiology[All Fields] OR childcare[All Fields] OR childcare'[All Fields] OR childcare's[All Fields] OR childcare1the[All Fields] OR childcarers[All Fields] OR childcarers'[All Fields] OR childcaring[All Fields] OR childcenter[All Fields] OR childcentred[All Fields] OR childchood[All Fields] OR childcognition[All Fields] OR childcom[All Fields] OR childcoun[All Fields] OR childd[All Fields] OR childdagger[All Fields] OR childdata[All Fields] OR childdeath[All Fields] OR childdecode[All Fields] OR childdemyelination[All Fields] OR childdevelopment[All Fields] OR childdgn[All Fields] OR childdgu[All Fields] OR childdhood[All Fields] OR childdoctor[All Fields] OR childdreams[All Fields] OR childdren[All Fields] OR childdren's[All Fields] OR childdynha[All Fields] OR childe[All Fields] OR childebayeva[All Fields] OR childebr[All Fields] OR childebran[All Fields] OR childebrand[All Fields] OR childed[All Fields] OR childeen[All Fields] OR childemi[All Fields] OR childen[All Fields] OR childen's[All Fields] OR childend[All Fields] OR childensmemorial[All Fields] OR childent[All Fields] OR childer[All Fields] OR childeren[All Fields] OR childeren's[All Fields] OR childerh[All Fields] OR childerhose[All Fields] OR childerhouse[All Fields] OR childeric[All Fields] OR childerick[All Fields] OR childerie[All Fields] OR childern[All Fields] OR childern's[All Fields] OR childerns[All Fields] OR childernsnational[All Fields] OR childerr[All Fields] OR childers[All Fields] OR childers'[All Fields] OR childersjw2[All Fields] OR childersllc[All Fields] OR childersmk[All Fields] OR childerson[All Fields] OR childerstone[All Fields] OR childes[All Fields] OR childesigns[All Fields] OR childester[All Fields] OR childevrepen[All Fields] OR childew[All Fields] OR childeyecare[All Fields] OR childfamilyconnections[All Fields] OR childfeeding[All Fields] OR childfile[All Fields] OR childfocused[All Fields] OR childfood[All Fields] OR childfree[All Fields] OR childfrom[All Fields] OR childfund[All Fields] OR childgood[All Fields] OR childgrowth[All Fields] OR childguidance[All Fields] OR childh[All Fields] OR childhaven's[All Fields] OR childhcod[All Fields] OR childhead[All Fields] OR childhealth[All Fields] OR childhealthcare[All Fields] OR childhealthresearch[All Fields] OR childhelp[All Fields] OR childhelp's[All Fields] OR childhhod[All Fields] OR childhhood[All Fields] OR childhod[All Fields] OR childhodd[All Fields] OR childhoiod[All Fields] OR childhold[All Fields] OR childhon[All Fields] OR childhoo[All Fields] OR childhood[All Fields] OR childhood'[All Fields] OR childhood''[All Fields] OR childhood'all[All Fields] OR childhood's[All Fields] OR childhood,[All Fields] OR childhood2000[All Fields] OR childhood2010[All Fields] OR childhoodacute[All Fields] OR childhoodadolescent[All Fields] OR childhoodcancer[All Fields] OR childhooddiseases[All Fields] OR childhoodfrom[All Fields] OR childhoodheadache[All Fields] OR childhoodobesity[All Fields] OR childhoodpulmonary[All Fields] OR childhoodreport[All Fields] OR childhoods[All Fields] OR childhoodtraumatic[All Fields] OR childhoodwas[All Fields] OR childhoodwith[All Fields] OR childhoof[All Fields] OR childhoofd[All Fields] OR childhool[All Fields] OR childhoold[All Fields] OR childhoond[All Fields] OR childhoood[All Fields] OR childhoos[All Fields] OR childhoot[All Fields] OR childhope[All Fields] OR childhosp[All Fields] OR childhospice[All Fields] OR childhours[All Fields] OR childhren[All Fields] OR childi[All Fields] OR childia[All Fields] OR childiae[All Fields] OR childiaeva[All Fields] OR childibaev[All Fields] OR childichimo[All Fields] OR childiidae[All Fields] OR childinfo[All Fields] OR childiren's[All Fields] OR childis[All Fields] OR childisch[All Fields] OR childish[All Fields] OR childish'[All Fields] OR childishexpert[All Fields] OR childishly[All Fields] OR childishness[All Fields] OR childism[All Fields] OR childist[All Fields] OR childkind[All Fields] OR childlaw[All Fields] OR childlen[All Fields] OR childless[All Fields] OR childless'[All Fields] OR childlessness[All Fields] OR childlessness'[All Fields] OR childlex[All Fields] OR childlhood[All Fields] OR childlight[All Fields] OR childlike[All Fields] OR childlikeness[All Fields] OR childline[All Fields] OR childline's[All Fields] OR childliver[All Fields] OR childloss[All Fields] OR childlove[All Fields] OR childlren's[All Fields] OR childm[All Fields] OR childmed[All Fields] OR childmedodallas[All Fields] OR childmeds[All Fields] OR childmind[All Fields] OR childminder[All Fields] OR childminder's[All Fields] OR childminders[All Fields] OR childminders'[All Fields] OR childminding[All Fields] OR childmother[All Fields] OR childneph[All Fields] OR childness[All Fields] OR childnessness[All Fields] OR childnet[All Fields] OR childneuro[All Fields] OR childneurology[All Fields] OR childneurologysociety[All Fields] OR childneuropsychiatry[All Fields] OR childneuropsychology[All Fields] OR childnood[All Fields] OR childnr[All Fields] OR childnurse[All Fields] OR childobesity[All Fields] OR childobesity180[All Fields] OR childoc[All Fields] OR childoflebanon[All Fields] OR childomics[All Fields] OR childonium[All Fields] OR childood[All Fields] OR childoriented[All Fields] OR childparent[All Fields] OR childpersonality[All Fields] OR childplay[All Fields] OR childprev[All Fields] OR childproof[All Fields] OR childproofed[All Fields] OR childproofing[All Fields] OR childproofing'[All Fields] OR childprotective[All Fields] OR childpsy8[All Fields] OR childpsych[All Fields] OR childpsychiatric[All Fields] OR childpsychiatrist[All Fields] OR childpsychiatrists[All Fields] OR childpsychiatry[All Fields] OR childpsychlaw[All Fields] OR childpsychology[All Fields] OR childpsychopharmacologyinstitute[All Fields] OR childpsychotherapeutic[All Fields] OR childpsychresearch[All Fields] OR childptsd[All Fields] OR childpugh[All Fields] OR childr[All Fields] OR childraising[All Fields] OR childrden's[All Fields] OR childre[All Fields] OR childre's[All Fields] OR childreach[All Fields] OR childrean[All Fields] OR childrearers[All Fields] OR childrearing[All Fields] OR childrearing'[All Fields] OR childred[All Fields] OR childredn[All Fields] OR childree[All Fields] OR childreen[All Fields] OR childrelationships[All Fields] OR childrem[All Fields] OR children[All Fields] OR children'[All Fields] OR children''[All Fields] OR children''s[All Fields] OR children'a[All Fields] OR children'car[All Fields] OR children'emotional[All Fields] OR children'hospital[All Fields] OR children'mc[All Fields] OR children'pital[All Fields] OR children'rights[All Fields] OR children's[All Fields] OR children's'[All Fields] OR children's'hands[All Fields] OR children'scancer[All Fields] OR children'scatalgine[All Fields] OR children'sclinic[All Fields] OR children'shospital[All Fields] OR children'significantly[All Fields] OR children'smedical[All Fields] OR children'smemorial[All Fields] OR children'snational[All Fields] OR children'sresearch[All Fields] OR children't[All Fields] OR children,[All Fields] OR children1[All Fields] OR children25[All Fields] OR children3[All Fields] OR children4hero[All Fields] OR children7[All Fields] OR childrena[All Fields] OR childrena'a's[All Fields] OR childrenae[All Fields] OR childrenaged[All Fields] OR childrenand[All Fields] OR childrenas[All Fields] OR childrenat[All Fields] OR childrenbristol[All Fields] OR childrenbychoice[All Fields] OR childrencentre1[All Fields] OR childrencompared[All Fields] OR childrend's[All Fields] OR childrendagger[All Fields] OR childrendaggerrocky[All Fields] OR childrendata[All Fields] OR childrendharvard[All Fields] OR childrendiagnosis[All Fields] OR childrendisease[All Fields] OR childrenduring[All Fields] OR childrenes[All Fields] OR childrenese[All Fields] OR childreneyecare[All Fields] OR childrenfirst[All Fields] OR childrenfrom[All Fields] OR childrenfs[All Fields] OR childrengammas[All Fields] OR childrenhad[All Fields] OR childrenhandskills[All Fields] OR childrenhood[All Fields] OR childrenhospital[All Fields] OR childrenhs[All Fields] OR childreni[All Fields] OR childrenii[All Fields] OR childrenin[All Fields] OR childrenindiana[All Fields] OR childreninterview[All Fields] OR childrenis[All Fields] OR childrenlondonukthe[All Fields] OR childrenmemorial[All Fields] OR childrenmn[All Fields] OR childrenmore[All Fields] OR childrenn[All Fields] OR childrenn's[All Fields] OR childrennhs[All Fields] OR childrennow[All Fields] OR childrenns[All Fields] OR childrenof[All Fields] OR childrenoncologygroup[All Fields] OR childrenos[All Fields] OR childrenover[All Fields] OR childrenpediatric[All Fields] OR childrenpen[All Fields] OR childrenperth[All Fields] OR childrenrial[All Fields] OR childrenrsquo[All Fields] OR childrenrsquos[All Fields] OR childrens[All Fields] OR childrens'[All Fields] OR childrens's[All Fields] OR childrens99[All Fields] OR childrensacramento[All Fields] OR childrensaidsociety[All Fields] OR childrensal[All Fields] OR childrensational[All Fields] OR childrensboard[All Fields] OR childrensbrain[All Fields] OR childrenscause[All Fields] OR childrenscentalcal[All Fields] OR childrenscentracal[All Fields] OR childrenscentral[All Fields] OR childrenscentralcal[All Fields] OR childrenscentre[All Fields] OR childrensch[All Fields] OR childrenscolorado[All Fields] OR childrensdayton[All Fields] OR childrensdefense[All Fields] OR childrensent[All Fields] OR childrensfoodtrust[All Fields] OR childrenshc[All Fields] OR childrensheartcenter[All Fields] OR childrenshospice[All Fields] OR childrenshospital[All Fields] OR childrenshospitals[All Fields] OR childrensi[All Fields] OR childrensinstitute[All Fields] OR childrensintitute[All Fields] OR childrenslawgroup[All Fields] OR childrenslighthousemn[All Fields] OR childrensmall[All Fields] OR childrensmemeorial[All Fields] OR childrensmemorail[All Fields] OR childrensmemorial[All Fields] OR childrensmn[All Fields] OR childrensnational[All Fields] OR childrensnationalmedicalcenter[All Fields] OR childrensocolorado[All Fields] OR childrensomaha[All Fields] OR childrensoncology[All Fields] OR childrensoncologygroup[All Fields] OR childrenspainguideline[All Fields] OR childrenspg[All Fields] OR childrenss[All Fields] OR childrensurology[All Fields] OR childrensuspected[All Fields] OR childrensustaining[All Fields] OR childrentitle[All Fields] OR childrentm[All Fields] OR childrentoronto[All Fields] OR childrentorontoontariocanada[All Fields] OR childrentorontoontariocanadam5g[All Fields] OR childrenuniversity[All Fields] OR childrenvillage[All Fields] OR childrenwas[All Fields] OR childrenweighing[All Fields] OR childrenwere[All Fields] OR childrenwhen[All Fields] OR childrenwho[All Fields] OR childrenwilmington[All Fields] OR childrenwith[All Fields] OR childrenwithin[All Fields] OR childrenx00b4[All Fields] OR childrenxmedian[All Fields] OR childrenxs[All Fields] OR childrepresents[All Fields] OR childreq[All Fields] OR childrerl[All Fields] OR childrern[All Fields] OR childrern's[All Fields] OR childrerns[All Fields] OR childres[All Fields] OR childres's[All Fields] OR childresn's[All Fields] OR childress[All Fields] OR childress'[All Fields] OR childress's[All Fields] OR childressi[All Fields] OR childressness[All Fields] OR childrestone[All Fields] OR childrestrains[All Fields] OR childreth[All Fields] OR childreti[All Fields] OR childrey[All Fields] OR childrhood[All Fields] OR childrive[All Fields] OR childrn[All Fields] OR childrn's[All Fields] OR childrne[All Fields] OR childrne's[All Fields] OR childrnen's[All Fields] OR childroom[All Fields] OR childrren[All Fields] OR childrren's[All Fields] OR childs[All Fields] OR childs'[All Fields] OR childs'b[All Fields] OR childs2[All Fields] OR childsa[All Fields] OR childsafe[All Fields] OR childsafetyeurope[All Fields] OR childsaving[All Fields] OR childscore[All Fields] OR childself[All Fields] OR childseq[All Fields] OR childserv[All Fields] OR childserve[All Fields] OR childsgv[All Fields] OR childsgwenv[All Fields] OR childshaheen[All Fields] OR childsight[All Fields] OR childsii[All Fields] OR childsish[All Fields] OR childsitters[All Fields] OR childsjd[All Fields] OR childsmile[All Fields] OR childsmile's[All Fields] OR childspacing[All Fields] OR childspla[All Fields] OR childsplay[All Fields] OR childspsych[All Fields] OR childsr[All Fields] OR childsubtotal[All Fields] OR childtending[All Fields] OR childtrauma[All Fields] OR childtrends[All Fields] OR childuplift[All Fields] OR childurology[All Fields] OR childwall[All Fields] OR childwards[All Fields] OR childwatch[All Fields] OR childways[All Fields] OR childwelfare[All Fields] OR childwinnie[All Fields] OR childwise[All Fields] OR childwiseresources[All Fields] OR childwish[All Fields] OR childwood[All Fields] OR childx[All Fields] OR childz[All Fields] OR childzen's[All Fields]) OR (school[All Fields] OR school'[All Fields] OR school''[All Fields] OR school's[All Fields] OR school1[All Fields] OR school1301[All Fields] OR school1981[All Fields] OR school2[All Fields] OR school2center[All Fields] OR school2division[All Fields] OR school3department[All Fields] OR school3division[All Fields] OR school55[All Fields] OR school79[All Fields] OR school8[All Fields] OR schoolability[All Fields] OR schoolage[All Fields] OR schoolaged[All Fields] OR schoolager's[All Fields] OR schoolagers[All Fields] OR schoolagers'[All Fields] OR schoolamerican[All Fields] OR schoolamps[All Fields] OR schooland[All Fields] OR schoolann[All Fields] OR schoolar[All Fields] OR schoolares[All Fields] OR schoolarity[All Fields] OR schoolarized[All Fields] OR schoolars[All Fields] OR schoolarts[All Fields] OR schoolartsendienst[All Fields] OR schoolartsendistrict[All Fields] OR schoolasthmaallergy[All Fields] OR schoolat[All Fields] OR schoolatbeth[All Fields] OR schoolathens[All Fields] OR schoolattendance[All Fields] OR schoolbag[All Fields] OR schoolbags[All Fields] OR schoolbased[All Fields] OR schoolbdepartment[All Fields] OR schoolbdivision[All Fields] OR schoolbeat[All Fields] OR schoolbeginners[All Fields] OR schoolbergenstraat[All Fields] OR schoolberlingermany[All Fields] OR schoolbesmetting[All Fields] OR schoolbeth[All Fields] OR schoolbevolking[All Fields] OR schoolbook[All Fields] OR schoolbooks[All Fields] OR schoolboston[All Fields] OR schoolbostonmassachusetts[All Fields] OR schoolbostonmassachusetts02115[All Fields] OR schoolboy[All Fields] OR schoolboys[All Fields] OR schoolboys'[All Fields] OR schoolbread[All Fields] OR schoolbullying[All Fields] OR schoolbuniversity[All Fields] OR schoolbus[All Fields] OR schoolby[All Fields] OR schoolcarl[All Fields] OR schoolcases[All Fields] OR schoolccornwall[All Fields] OR schoolcenter[All Fields] OR schoolcharles[All Fields] OR schoolcharlestown[All Fields] OR schoolcharlestownmassachusetts[All Fields] OR schoolchidren[All Fields] OR schoolchild[All Fields] OR schoolchild's[All Fields] OR schoolchilden[All Fields] OR schoolchilderen[All Fields] OR schoolchildred[All Fields] OR schoolchildren[All Fields] OR schoolchildren'[All Fields] OR schoolchildren's[All Fields] OR schoolchildrens[All Fields] OR schoolchildrens'[All Fields] OR schoolchildresn[All Fields] OR schoolchldren[All Fields] OR schoolcijfers[All Fields] OR schoolclass[All Fields] OR schoolclasses[All Fields] OR schoolcolumbia[All Fields] OR schoolcraft[All Fields] OR schoolcraft's[All Fields] OR schoold[All Fields] OR schooldaggeralbert[All Fields] OR schooldaggerdepartment[All Fields] OR schooldaggermodeling[All Fields] OR schooldaggeruniversity[All Fields] OR schooldanga[All Fields] OR schoolday[All Fields] OR schooldays[All Fields] OR schoolden[All Fields] OR schooldepartment[All Fields] OR schoolderman[All Fields] OR schooldermann[All Fields] OR schooldesks[All Fields] OR schooldirector[All Fields] OR schooldoctors[All Fields] OR schooldog[All Fields] OR schooldouble[All Fields] OR schooldundee[All Fields] OR schoole[All Fields] OR schoolec[All Fields] OR schooled[All Fields] OR schooled'[All Fields] OR schooledepartment[All Fields] OR schooleducation[All Fields] OR schoolegebeuren[All Fields] OR schooleman[All Fields] OR schoolengland[All Fields] OR schooler[All Fields] OR schooler's[All Fields] OR schoolers[All Fields] OR schoolers'[All Fields] OR schoolery[All Fields] OR schooley[All Fields] OR schooley's[All Fields] OR schoolf[All Fields] OR schoolfamily[All Fields] OR schoolfeeding[All Fields] OR schoolfellow[All Fields] OR schoolfellows[All Fields] OR schoolfield[All Fields] OR schoolflorence[All Fields] OR schoolfoodtrust[All Fields] OR schoolfor[All Fields] OR schoolfriend[All Fields] OR schoolfruit[All Fields] OR schoolgaan[All Fields] OR schoolgaande[All Fields] OR schoolgaanden[All Fields] OR schoolgebouw[All Fields] OR schoolgeneeskunde[All Fields] OR schoolgezondheidszorg[All Fields] OR schoolgirl[All Fields] OR schoolgirls[All Fields] OR schoolgirls'[All Fields] OR schoolgoi[All Fields] OR schoolgoing[All Fields] OR schoolgrade[All Fields] OR schoolgrades[All Fields] OR schoolgraduates[All Fields] OR schoolgroup[All Fields] OR schoolgruiten[All Fields] OR schoolgwangju[All Fields] OR schoolhannover[All Fields] OR schoolhealthcenters[All Fields] OR schoolhealthlink[All Fields] OR schoolhill[All Fields] OR schoolhomburg[All Fields] OR schoolhood[All Fields] OR schoolhouse[All Fields] OR schoolhouses[All Fields] OR schoolhouston[All Fields] OR schoolhygien[All Fields] OR schoolhygiene[All Fields] OR schoolies[All Fields] OR schoolies'[All Fields] OR schoolin[All Fields] OR schoolinanjing[All Fields] OR schooling[All Fields] OR schooling'[All Fields] OR schooling's[All Fields] OR schoolings[All Fields] OR schoolinnsbruck[All Fields] OR schoolinstitute[All Fields] OR schoolinzai[All Fields] OR schooliosis[All Fields] OR schooljeonju[All Fields] OR schooljerusalem[All Fields] OR schooljeugd[All Fields] OR schoolkantines[All Fields] OR schoolkawachi[All Fields] OR schoolkids[All Fields] OR schoolkind[All Fields] OR schoolkindereen[All Fields] OR schoolkinderen[All Fields] OR schoolkinderonderzoek[All Fields] OR schoolkindersterfte[All Fields] OR schoolkolling[All Fields] OR schooll[All Fields] OR schoolland[All Fields] OR schoolleaving[All Fields] OR schoolleerling[All Fields] OR schoolljubljana[All Fields] OR schoollondon[All Fields] OR schoolloopbaan[All Fields] OR schoollunch[All Fields] OR schoolm[All Fields] OR schoolmaatschappelijk[All Fields] OR schoolmadrid[All Fields] OR schoolman[All Fields] OR schoolmanchester[All Fields] OR schoolmann[All Fields] OR schoolmark[All Fields] OR schoolmarks[All Fields] OR schoolmarm[All Fields] OR schoolmarmish[All Fields] OR schoolmaster[All Fields] OR schoolmaster's[All Fields] OR schoolmasters[All Fields] OR schoolmasters'[All Fields] OR schoolmatch[All Fields] OR schoolmate[All Fields] OR schoolmate's[All Fields] OR schoolmater[All Fields] OR schoolmates[All Fields] OR schoolmates'[All Fields] OR schoolmeals[All Fields] OR schoolmeester[All Fields] OR schoolmeesters[All Fields] OR schoolmelbourne[All Fields] OR schoolmelk[All Fields] OR schoolmen[All Fields] OR schoolmerich[All Fields] OR schoolmiami[All Fields] OR schoolmilwaukee[All Fields] OR schoolminneapolis[All Fields] OR schoolmist[All Fields] OR schoolmistresses[All Fields] OR schoolnagoya[All Fields] OR schoolnanjing[All Fields] OR schoolnankoku[All Fields] OR schoolnewarknjusa[All Fields] OR schoolni[All Fields] OR schoolnick[All Fields] OR schoolnik[All Fields] OR schoolo[All Fields] OR schoolof[All Fields] OR schoolofmedicine[All Fields] OR schoolofsurgery[All Fields] OR schoology[All Fields] OR schoolopleiding[All Fields] OR schoolov[All Fields] OR schoolpeking[All Fields] OR schoolph[All Fields] OR schoolphobic[All Fields] OR schoolphobie[All Fields] OR schoolpopulatie[All Fields] OR schoolporto[All Fields] OR schoolprestaties[All Fields] OR schoolproblemen[All Fields] OR schoolpupils[All Fields] OR schoolr[All Fields] OR schoolrendement[All Fields] OR schoolresearch[All Fields] OR schoolribeirao[All Fields] OR schoolrijpheid[All Fields] OR schoolrik[All Fields] OR schoolrochester[All Fields] OR schoolroom[All Fields] OR schoolrooms[All Fields] OR schools[All Fields] OR schools'[All Fields] OR schools1[All Fields] OR schoolsafety[All Fields] OR schoolsao[All Fields] OR schoolschildren[All Fields] OR schoolschneeweiss[All Fields] OR schoolschool[All Fields] OR schoolschool2[All Fields] OR schoolservice[All Fields] OR schoolshave[All Fields] OR schoolshcildren[All Fields] OR schoolshealth[All Fields] OR schoolshenzhen[All Fields] OR schoolshenzhenguangdong518055china[All Fields] OR schoolshriners[All Fields] OR schoolsingapore[All Fields] OR schoolsite[All Fields] OR schoolsluiting[All Fields] OR schoolsofnursingscrutinized[All Fields] OR schoolspace[All Fields] OR schoolspace'[All Fields] OR schoolstarters[All Fields] OR schoolsthe[All Fields] OR schoolstraat[All Fields] OR schoolstudents[All Fields] OR schoolstudents'[All Fields] OR schoolswiss[All Fields] OR schooltaipeitaiwan[All Fields] OR schooltandartsentekort[All Fields] OR schooltandonderzoek[All Fields] OR schooltandverzorging[All Fields] OR schooltandverzorgsters[All Fields] OR schoolteacher[All Fields] OR schoolteacher's[All Fields] OR schoolteachers[All Fields] OR schoolteachers'[All Fields] OR schoolteaching[All Fields] OR schoolthe[All Fields] OR schoolthessalonikigreece54124[All Fields] OR schooltime[All Fields] OR schooltink[All Fields] OR schooltoezicht[All Fields] OR schooltokyo[All Fields] OR schoolts[All Fields] OR schooltubingengermany[All Fields] OR schooltypes[All Fields] OR schooluitslagen[All Fields] OR schooluniversity[All Fields] OR schoolvanderbilt[All Fields] OR schoolverona[All Fields] OR schoolverpleegkundige[All Fields] OR schoolverpleegkundigen[All Fields] OR schoolverzuim[All Fields] OR schoolvoeding[All Fields] OR schoolvoorbeeld[All Fields] OR schoolvorderingen[All Fields] OR schoolvorderlingen[All Fields] OR schoolwear[All Fields] OR schoolwerkplanontwikkeling[All Fields] OR schoolwerth[All Fields] OR schoolwide[All Fields] OR schoolwise[All Fields] OR schoolworcester[All Fields] OR schoolwork[All Fields] OR schoolwork'[All Fields] OR schoolyard[All Fields] OR schoolyards[All Fields] OR schoolyear[All Fields] OR schoolziekte[All Fields]) OR (adolescen[All Fields] OR adolescenata[All Fields] OR adolescence[All Fields] OR adolescence'[All Fields] OR adolescence's[All Fields] OR adolescence,[All Fields] OR adolescenceadulthood[All Fields] OR adolescencecan[All Fields] OR adolescencents[All Fields] OR adolescences[All Fields] OR adolescences'[All Fields] OR adolescencet[All Fields] OR adolescenci[All Fields] OR adolescencia[All Fields] OR adolescencie[All Fields] OR adolescencii[All Fields] OR adolescencija[All Fields] OR adolescenciji[All Fields] OR adolescencji[All Fields] OR adolescenct[All Fields] OR adolescency[All Fields] OR adolescene[All Fields] OR adolescenies[All Fields] OR adolescens[All Fields] OR adolescense[All Fields] OR adolescensen[All Fields] OR adolescensmedicin[All Fields] OR adolescent[All Fields] OR adolescent'[All Fields] OR adolescent'psychosocial[All Fields] OR adolescent's[All Fields] OR adolescent0288[All Fields] OR adolescenta[All Fields] OR adolescentadult[All Fields] OR adolescentaids[All Fields] OR adolescentaire[All Fields] OR adolescentclinic[All Fields] OR adolescentdevelopment[All Fields] OR adolescente[All Fields] OR adolescente'[All Fields] OR adolescentei[All Fields] OR adolescenten[All Fields] OR adolescentenalter[All Fields] OR adolescentencriminaliteit[All Fields] OR adolescentenkliniek[All Fields] OR adolescentenleeftijd[All Fields] OR adolescentes[All Fields] OR adolescentes'[All Fields] OR adolescenthealth[All Fields] OR adolescenthealthlaw[All Fields] OR adolescenthood[All Fields] OR adolescenti[All Fields] OR adolescential[All Fields] OR adolescentica[All Fields] OR adolescentics[All Fields] OR adolescentie[All Fields] OR adolescentieleeftijd[All Fields] OR adolescentii[All Fields] OR adolescentilor[All Fields] OR adolescentis[All Fields] OR adolescentium[All Fields] OR adolescentkinja[All Fields] OR adolescentl[All Fields] OR adolescentne[All Fields] OR adolescentni[All Fields] OR adolescentnich[All Fields] OR adolescentnih[All Fields] OR adolescentnim[All Fields] OR adolescentno[All Fields] OR adolescentnog[All Fields] OR adolescentnoj[All Fields] OR adolescentnom[All Fields] OR adolescentnu[All Fields] OR adolescentnych[All Fields] OR adolescentologia[All Fields] OR adolescentologica[All Fields] OR adolescentological[All Fields] OR adolescentologico[All Fields] OR adolescentologie[All Fields] OR adolescentologist[All Fields] OR adolescentologo[All Fields] OR adolescentology[All Fields] OR adolescentology's[All Fields] OR adolescentov[All Fields] OR adolescentow[All Fields] OR adolescentpregnancy[All Fields] OR adolescentro[All Fields] OR adolescents[All Fields] OR adolescents'[All Fields] OR adolescents'ability[All Fields] OR adolescents'compliance[All Fields] OR adolescents'depression[All Fields] OR adolescents'dietary[All Fields] OR adolescents'distress[All Fields] OR adolescents'experiences[All Fields] OR adolescents'health[All Fields] OR adolescents'ill[All Fields] OR adolescents'intentions[All Fields] OR adolescents'lives[All Fields] OR adolescents'perceptions[All Fields] OR adolescents'physical[All Fields] OR adolescents'problem[All Fields] OR adolescents'psychosocial[All Fields] OR adolescents's[All Fields] OR adolescents'self[All Fields] OR adolescents'sensation[All Fields] OR adolescents'smoking[All Fields] OR adolescents'therapeutic[All Fields] OR adolescents,[All Fields] OR adolescentscopyright[All Fields] OR adolescentsdrk[All Fields] OR adolescentshealth[All Fields] OR adolescentsjeunes[All Fields] OR adolescentsoulu[All Fields] OR adolescentswith[All Fields] OR adolescentt[All Fields] OR adolescentu[All Fields] OR adolescentul[All Fields] OR adolescentului[All Fields] OR adolescentum[All Fields] OR adolescenty[All Fields] OR adolescenz[All Fields] OR adolescenza[All Fields] OR adolescenziale[All Fields] OR adolescenziali[All Fields]))
